# Multiscale Modeling of the RESET Sweep in a Single-Layer MoS_2_ Atomristor Using Density Functional Theory

**DOI:** 10.1021/acsomega.6c02926

**Published:** 2026-05-28

**Authors:** Aykut Turfanda, Alessio Gagliardi

**Affiliations:** † Department of Electrical Engineering, TUM School of Computation, Information and Technology, 9184 Technical University of Munich, Hans-Piloty-Straße 1, 85748 Garching, Germany; ‡ Atomistic Modeling Center (AMC) and Munich Data Science Institute (MDSI), 9184 Technical University of Munich, 85748 Garching, Germany

## Abstract

We study and model the physical mechanisms of the single-layer MoS_2_-based atomristor’s bipolar resistive switching at the RESET sweep. We aim to understand the operation of these thin devices to better optimize them in terms of energy consumption and endurance characteristics. We use density functional theory methods in concordance with analytical models and experimental results from the literature. We conclude that the mechanisms are related to trap characteristics and their fillings together with charge carrier relaxation in our self-consistent description of the operation. This work is important as it opens the way to optimize the performance of the atomristor by decreasing power dissipation and increasing the number of operation cycles.

## Graphical abstract

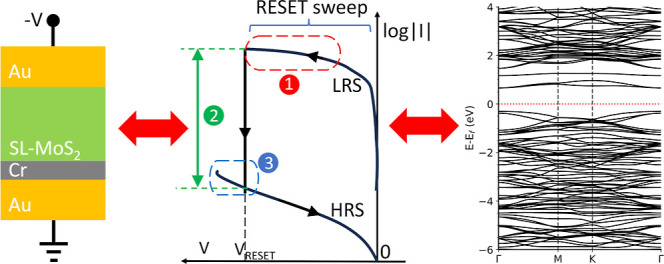

## Introduction

Resistive switching is a phenomenon mainly based on varying electric resistivity between at least two distinguishable states across an energy barrier under external effects, such as an applied voltage bias, in a reversible manner.^1^ In the realm of resistive switching, we model and study the RESET phenomenon of bipolar resistive switching in a two-terminal vertical device architecture with an active medium composed of an atomically thin single-layer MoS_2_. This device, called a single-layer MoS_2_-based atomristor, will be termed the atomristor, and the paper introducing it will be termed the atomristor article in this study.^2^

There are numerous operation principles and the abundant different device physics behind the resistive switching phenomena. Moreover, resistive switching devices exhibits high variability and low yields, even though a recent study suggests that “unpredicted variations” are necessary in switching for cryptography.^3^ Nonetheless, atomically thin resistive switching devices, like single-layer MoS_2_-based atomristors, are in focus for the microelectronics industry due to their advantages in low power consumption.^2,4^ Therefore, we model and investigate the operating principle of the atomristor and we simulate it using density functional theory, DFT, and multiscale simulations in accordance with analytical models and experimental results to better understand the operation of the atomristor in terms of its resistive switching parameters that are important for power consumption: RESET voltage, *V*_RESET_, and current at the RESET voltage. The importance of modeling is also revealed in a recent review article as relating the main limitations of the neuromorphic devices to the lack of their models compared to digital electronics.^5^ Moreover, to facilitate the atomristor’s integration into nextgeneration electronics in the age of artificial intelligence, we also need to predict the endurance performance and cycle-to-cycle variability.

RESET is important to investigate because its voltage and current values determine the power consumption of the atomristor. Therefore, a good understanding of RESET is more important than SET in terms of power consumption; even though the SET process directly affects the RESET in an atomristor in a cyclic manner. Nonetheless, these devices are not only used for low-power-consuming applications but also for highly adaptive applications because these devices are defected systems and they allow the easy control of charge carriers (carrier type and carrier density), which makes them a platform to serve as trapping centers for the charge carriers.^6^ For example, an atomically thin material-based channel is used to create a dynamically reconfigurable memory device in the literature.^7^ Similarly, controlling these defect dynamics and charge carriers in atomristors is important for the future of resistive switching devices and, most importantly, for use in brain-like computing. In this way, these modifications need to be understood well and modeled and quantified.

Atomically thin materials are sensitive to variations in the number of defects whether they are used in vertical two-terminal or field-effect transistor, FET, architectures; even though recent literature focuses on FETs with atomically thin materials serving as a channel.^8,9^ This high dependence of performance on the number of defects, their configurations and positions, and their charging characteristics in these devices is of utmost importance when we consider the single-layer MoS_2_-based atomristor.^10^ We know that the research done using this type of resistive switching device yields experimental results, such as current–voltage, *I*–*V*, characteristics, as shown in the atomristor article.^2^ We can compare these results with analytical models through computational studies because of the well-established Schottky junction theory.^11^ This investigation of a Schottky barrier is not only useful for two-terminal devices but also useful for the metal–semiconductor FETs and high-electron-mobility transistors because their gate electrodes are also Schottky junctions.

We study the Au electrode and the Au-doped single-layer MoS_2_ junction with computational methods based on analytical models together with density functional theory, DFT, to explain the RESET operation mechanism of the single-layer MoS_2_-based atomristor, which is similar to our investigation of the SET sweep in our so-called SET study in the literature.^12^ Unlike the existing literature on atomristors, which is primarily based on DFT simulations, our approach is distinctive in that it integrates experimental data from the literature with modeling and analysis. Moreover, we provide a step-by-step explanation of the resistive switching mechanism mainly driven by charged dopants with a particular emphasis on the RESET process that has been rarely explored in previous studies. We investigate the mode of electron transport during the RESET sweep of a single-layer MoS_2_-based atomristor, which is composed of (i) a low-resistive state before the RESET process, (ii) the RESET process, and (iii) a high-resistive state after the RESET process. The operation mechanism of the single-layer MoS_2_-based atomristors is important to further manipulate the devices to more closely resemble the characteristics of synapses and therefore to reduce power consumption and to increase endurance performance. The proposed mechanism can be considered as a physically motivated and self-consistent explanation of atomristors, rather than a definitive detection of the microscopic and dynamic resistive switching process.

## Computational Methods

Crystallographic information files, CIF, of bulk MoS_2_, MoSe_2_, WS_2_, and WSe_2_, were downloaded from the materials project Web site.^13^ Then, single layers are obtained as explained in the computational methods section of the SET study.^12^ Geometry optimization and electronic structure determination calculations are performed in Quantum ESPRESSO, version 7.0.^14–16^ All parameters used in simulations are kept the same as the computational methods section of the SET study, including *k*-points and pseudopotentials.^17^ We use the Perdew–Burke–Ernzerhof (PBE) functional and the pseudopotentials used are SG15 optimized norm-conserving Vanderbilt scalar-relativistic pseudopotentials for electrostatic potential energy calculations and for geometry optimization of structures.^18–22^ For the electronic structure calculations, we use optimized norm-conserving Vanderbilt pseudopotentials from Pseudo Dojo to be compatible with the SET study.^22,23^ We consider 160 DFT bands for Au-doped (3 × 3) single-layer MoS_2_’s electronic band structure both in its charged configurations and under electric field. The mentioned electric field during the band structure studies is applied to (3 × 3) supercells with a sawtooth-like electronic potential, where 1 atomic unit (au) is equal to 51.42 × 10^10^ V·m^–1^. We obtain the band structures with electric field without performing a geometry relaxation under the electric field.

Electrostatic potential energy is calculated using Quantum ESPRESSO’s input variable, plot_num = 11, for Au-doped (3 × 3) single-layer MoS_2_, which represents the bare potential and the Hartree potential. Gold-doped neutral and positively charged (3 × 3) supercells of MoS_2_ single layers are relaxed (atomic positions are optimized by keeping the lattice constant fixed) using the same parameters that we used for the geometry optimization of the single-layer MoS_2_ unit cell (for the details see the SET study), except for the Monkhorst–Pack grid of (6 × 6 × 1). In our study, positively charged and negatively charged supercells mean that the calculation is performed using the Quantum ESPRESSO’s tot_charge input variable. Finally, energy corrections in defect formation energy diagrams from the literature were obtained using sxdefectaligner2d software and VESTA is used for visualization.^24,25^ We did not use assume_isolated = 2D input variable in any of our calculations, which is used for the truncation of Coulomb interaction. We ensure at least 18 Å of vacuum in the supercell model of single layer with a Au dopant. The main reference system is also depicted in Figure S1 of the Supporting Information.

Crystal orbital Hamilton population, COHP, analysis is carried out using the LOBSTER code (version 4.1.0), where the PAW-type pseudopotentials are used as given Au.pbe-spn-kjpaw_psl.1.0.0.UPF for Au, Mo.pbe-spn-kjpaw_psl.1.0.0.UPF for Mo, and S.pbe-n-kjpaw_psl.1.0.0.UPF for S with the recommended basis functions of 5d, 5p, 5s, 6s for Au; 4d, 4p, 4s, 5s for Mo; and 3p, 3s for S, and 800 Ry is taken as the plane-wave cutoff for charge density.^26–28^ Projected density of states (PDOS) calculations are performed using norm-conserving Vanderbilt pseudopotentials from Pseudo Dojo with a Monkhorst–Pack grid of (6 × 6 × 1) for self-consistent field calculations and (18 × 18 × 1) for the nonself-consistent field calculations with tetrahedral occupations and considering 180 DFT bands. Later, we plot PDOS using a Gaussian broadening (degauss) value of 0.005 Ry and an energy grid step of 0.01 eV. Then, we calculate the partial charge density for a certain energy range in real space and we visualize the contribution of a certain wave function to the charge density. Since we use norm-conserving pseudo potentials, this is |ψ|^2^, where ψ is the selected wave function. The postprocessing (pp.x) scripts for the visualization of partial charge density and |ψ|^2^ calculations are given in Section 1 of the Supporting Information. For details of the computational methods used in this article, please see the SET study.^12^

We also use SIESTA code to do DFT calculations.^29,30^ Here, exchange–correlation functional PBE is used. Norm-conserving pseudopotentials (Troullier-Martins) are used, while valence states are expanded using a double-ζ polarized (DZP) numerical atomic orbital basis set.^31^ The energy shift is set to 0.015 Ry and a mesh cutoff of 400 Ry is used. Electronic self-consistency calculations are performed using Pulay mixing with a mixing weight of 0.10 and eight Pulay steps. A dipole correction was applied perpendicular to the slab and external electric field was applied along the out-of-plane (*z*) direction.^32^ A net positive charge was introduced in the cell (in units of |*e*|) and doping was treated using compensating background charge. Structural relaxations in Siesta are performed using the conjugate gradient (CG) method, where only atomic positions are optimized until the forces on all atoms are below 0.05 eV/Å, and lattice vectors are kept fixed. Spin polarization is not included. For (5 × 5) supercell-based calculations, we use the Monkhorst–Pack grid of (3 × 3 × 1).

## Results and Discussion

### Low-Resistive State before the RESET Process

We investigate the I–V characteristics of the single-layer MoS_2_-based atomristor as it is depicted in Figure 1(a), with its top and bottom Au electrodes, Cr sticking layer, and single-layer MoS_2_, where the Au top electrode forms a high barrier for charge carriers, while the oxidizable Cr layer is considered to play a role analogous to Ti, providing an Ohmic interface, as commonly discussed in the literature for Ti-based contacts.^33,34^ We first recognize that this *I*–*V* characteristics pictured in Figure 1(b) is very similar to the type two conduction as given by Carsten Funck and Stephan Menzel, where they relate the resistive switching based on four main concepts, these are, trap-assisted tunneling, space-charge-limited current, Poole–Frenkel conduction, and Schottky barrier-limited transport.^33^ We inspired from this article and therefore we also benefit from some of these concepts either directly or in their modified forms in our explanation of single-layer MoS_2_-based atomristor’s resistive switching; however, that work was for bulk oxides and therefore, we also need to consider the atomic thickness of the atomically thin materials in our explanation using the methods of DFT.

**Figure 1:**
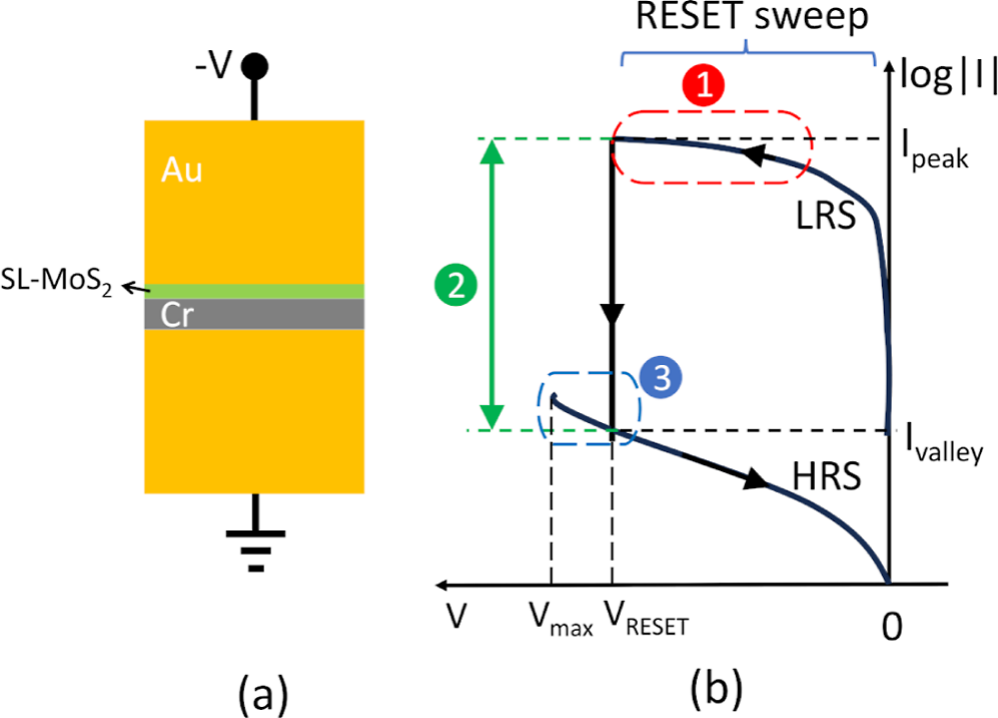
(a) A cartoon depicting a single-layer, SL, MoS_2_-based atomristor with Au electrodes and Cr layer (not to scale) and (b) a schematic drawing of the RESET sweep of the atomristor’s current–voltage, I–V, characteristics with its region 1, region 2, and region 3, which are enclosed by red, green, and blue dashed lines, respectively, from the literature. Low-resistive state is abbreviated as LRS, high-resistive state is abbreviated as HRS, V_RESET_ is the RESET voltage, *V*_max_ is the maximum voltage at RESET, and log|*I*| denotes the logarithmic axis. Adapted and redrawn with permission from *Nano Lett*. **2018**, 18, 1, 434–441 (ref 2). Copyright Ⓒ 2017 American Chemical Society.

As we discussed in the article in which we investigate the SET sweep of the atomristor, which will be referred to as the “SET study” throughout this article, we again consider the top Au electrode as an active electrode (not in the context of electrochemical devices but as a determinant electrode of resistive switching). As we discussed in the SET study, in SET, there was sulfur vacancy-based link formation, which we want to further support this “localized” channel assumption based on the atomristor article because low-resistive state’s resistance has almost no area dependence compared to the high-resistive state’s resistance at 0.1 V. Based on a current review article, this almost area independence of the low-resistive state indicates a localized switching mechanism.^5^ As discussed in the SET study, Au melts or evaporates and is eventually incorporated into the single-layer MoS_2_ due to local heating, where the Au concentration involved in each switching cycle is also influenced from this, and we model this melted or evaporated Au in single-layer MoS_2_ as a substitutional dopant in our DFT-based studies. Moreover, we rely on the thermal properties of single-layer MoS_2_ without considering its thermal boundary resistance with the Au electrode to find the number of sulfur vacancies to bring Au into the single-layer MoS_2_ after local heating. More precisely, we can consider the thermal boundary resistance when we calculate the number of links based on thermal conductance of single-layer MoS_2_.^35^ Nevertheless, this is out of scope of this article.

The mode of transport at the beginning of the RESET sweep (before region 1 in Figure 1(b)) is similar to the SET sweep after the SET process.^12^ This transport mode is Ohmic-like due to Au dopants, as discussed in the SET study. These Ohmic-like characteristics imply the dominance of tunneling. We can roughly check the dominance of tunneling (field emission) by testing whether our system fulfills the *kT* ≪ *E*_00_ relation or not, where *k* is the Boltzmann constant, *T* is the temperature, and *E*_00_ is defined as in eq 1,

1 $$E_{00}\equiv \frac{q\hbar }{2}\sqrt{\frac{N}{m_{h}^{*}\cdot \epsilon_{o}\cdot \epsilon_{2-D,⊥}}}$$

where *q* is the elementary electric charge, ℏ is the reduced Planck constant, *N* is the number density of Au incorporated into the single-layer MoS_2_ [m^–3^], *m*_h_^*^ is the hole effective mass in single-layer MoS_2_, ϵ_o_ is the vacuum permittivity, and ϵ_2‑D,⊥_ is the two-dimensional, 2-D, out-of-plane dielectric constant of single-layer MoS_2_.^36^ To set *N* in eq 1, we consult the SET study, where we claimed that we need 134 sulfur vacancies to bring one Au into single-layer MoS_2_ as a theoretical limit due to local heating as discussed in the SET study. This corresponds to a Au density of 7.4 × 10^9^ cm^–2^ in single-layer MoS_2_ after local heating. Nevertheless, we know that approximately two sulfur vacancies are needed to establish the conductive link. If we consider the latter case, where we need two sulfur vacancies to bring one Au into single-layer MoS_2_; namely, on the other side of the limit, we can have a Au density of 0.5 × 10^12^ cm^–2^ in single-layer MoS_2_ after local heating. Therefore, we depict *E*_00_ versus *N* relationship based on eq 1 in Figure 2 from *N* = 7.4 × 10^9^ cm^–2^ to *N* = 0.5 × 10^12^ cm^–2^. Then, we reach the so-called *kT* ≪ *E*_00_ regime when we consider that two sulfur vacancies are needed to bring Au into single-layer MoS_2_, as shown in Figure 2 at *T* = 300 K. This is reasonable due to the abundance of grain boundaries in the single-layer MoS_2_-based atomristor. This is also reasonable when we consider the multi cycle operation of the atomristor. Therefore, a higher *N* is possible; even though we neglect the temperature and defect concentration dependence of ϵ_2–D,⊥_. We also predict that not only the field emission (*kT* ≪ *E*_00_) but also the thermionic-field emission (*kT* ≈ *E*_00_) is the dominant type of electron transport just before region 1 in Figure 1, where *kT* ≪ *E*_00_ is marked with a red dashed line in Figure 2 based on eq 1.

**Figure 2:**
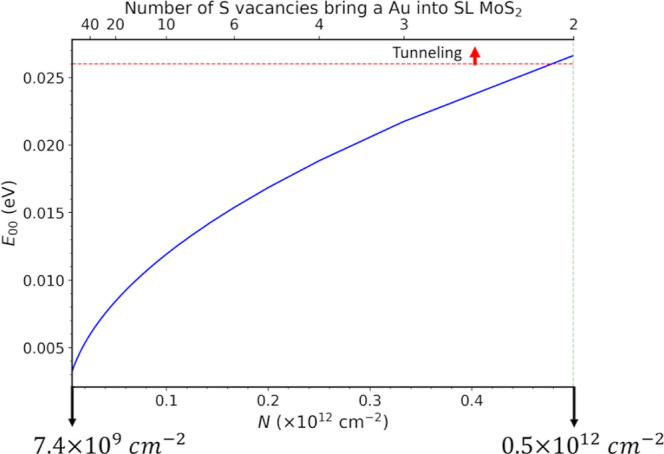
Relation of *E*_00_ versus the number density of Au dopants, *N* (replacing sulfur vacancies) in single-layer, SL, MoS_2_. Upper axis represents the number of sulfur vacancies needed to bring Au into single-layer MoS_2_, where *N* = 7.4 × 10^9^ cm^–2^ corresponds to a case, where 134 sulfur vacancies bring Au into single-layer MoS_2_ after local heating and *N* = 0.5 × 10^12^ cm^–2^ corresponds to a case where 2 sulfur vacancies bring Au into single-layer MoS_2_ after local heating. For *E*_00_ values above the red dashed line; namely, when 0.026 eV ≪ *E*_00_, we indicate the dominance of tunneling.

We quantitatively predict the electronic transport modes as thermionic emission and tunneling as concluded above based on *kT* ≈ *E*_00_ and *kT* ≪ *E*_00_. In the band structure picture of the Schottky barrier, we again investigate the type of transport at low-voltage values (from 0 V to −0.25 V), at the RESET for the low-resistive state. We therefore consider the system to be p-type prior to Au incorporation into single-layer MoS_2_ and treating Au as a cluster. Although our DFT model employs a single Au atom in a (3 × 3) supercell for computational tractability, the effective size and energetics are more representative of Au nanoparticle-like behavior. Previous studies indicate that while isolated Au adatoms may introduce midgap states and compensate n-type doping, larger Au clusters tend to induce p-type behavior; therefore, we have a forward-biased Schottky diode.^37–39^ In an ideal diode case, when the metal electrode is biased more negatively, the band bending of the semiconductor will decrease and holes will begin to transfer from the semiconductor side to the metal side and this current is mostly governed by thermionic emission. We consider that the Au dopants make single-layer MoS_2_ a heavily doped semiconductor locally. Therefore, for highly doped materials like the Au-doped single-layer MoS_2_ with a very high Schottky barrier for holes, tunneling of holes from the semiconductor to the metal; namely, the field emission through the very thin potential barrier (negatively biased metal and p-type semiconductor) at very low biases, is possible.^40^

We continue to investigate the remaining *I*–*V* characteristics in the low-resistive state of the RESET sweep toward the RESET voltage. To model and understand the *I*–*V* characteristics at high reverse voltage biases (from −0.25 V to −1.25 V; namely, region 1 in Figure 1(b)), we first consider the defect formation energy diagram of the Au-doped single-layer MoS_2_ as depicted in Figure 3, where we read the defect formation energy of the neutral state of the Au dopant as 3.18 eV. Similarly, we read the charge transition level (from −1 to 0) as 1.71 eV with respect to the valence band maximum, and we read the charge transition level (from 0 to +1) as 0.91 eV with respect to the valence band maximum. The formation energy of the charged defects is a function of the chemical potential energy. For example, the formation energy of the positively charged state of Au defect is lower than the neutral state of the Au defect as shown in Figure 3 if we consider the formation energies of the Au defects as depicted in Figure 3 and if we assume an efficiently decreasing chemical potential relative to the valence band maximum for the Au-doped single-layer MoS_2_ efficiently without Fermi-level pinning. The gold dopant might be charged because it is energetically more favorable than the neutral state.

**Figure 3:**
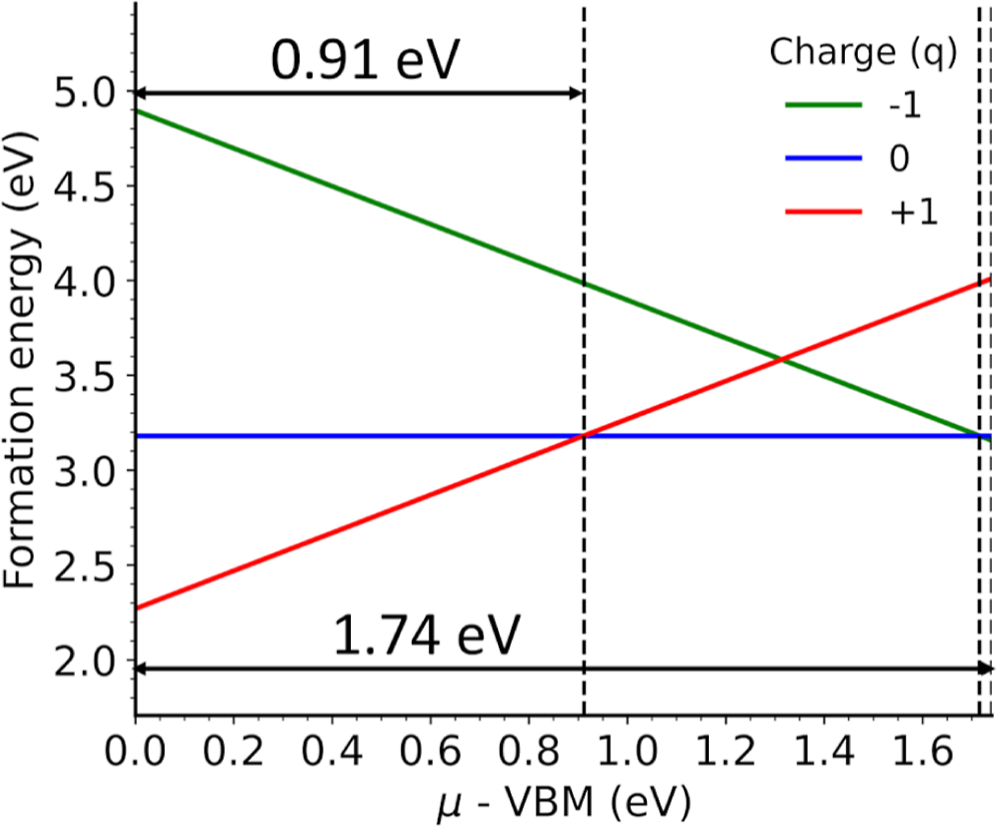
Defect formation energy diagram of Au-doped (3 × 3) single-layer MoS_2_ in −1q, 0, and +1q charge states, where q is the elementary electric charge, from the literature. The marked value of 1.74 eV represents the density functional theory band gap, while 0.91 eV represents the charge transition level from the +1q to 0 charge configuration with respect to the valence band maximum, VBM, where μ is the chemical potential. Adapted and redrawn from *ACS Appl. Electron. Mater*. 2025, **7**, 9, 3795–3809 (ref 12) under the Creative Commons Attribution 4.0 International License (CC-BY 4.0). Copyright Ⓒ 2025 The Authors. Published by the American Chemical Society.

The presence of a positively charged state of Au defect from the formation energy diagram as shown in Figure 3 implies that the Au dopant in this interval has donor-like characteristics. On the other hand, we need to consider the Au-doped single-layer MoS_2_ as it has a weak n-type tendency, rather than intrinsic n-type doping, but we will refer to this as an n-type semiconductor in this article, even though the exact determination of type is hard due to coexistence of many vacancies and dopants and due to varying number of defects and their varying charge characteristics during SET and RESET sweep cycles together with the effects of substrate. On the other hand, we neglect the formation of S vacancies during device operation, which can be another limiting factor for the n-type doping of single-layer MoS_2_, as discussed in the literature.^41^

In an ideal diode, under reverse bias, the metal Fermi level shifts upward (relative to semiconductor) and barrier becomes wider and bands bend at the semiconductor side. We expect to reach a so-called saturation current due to thermionic emission of electrons from metal to semiconductor, but we did not observe a saturation.^40^ Nevertheless, we can compare the current at a low-resistive state of the RESET at around *V* = −1.25 V from the atomristor article and we compare them as follows, $I_{MoS_{2}}^{at-1.25V}$ > $I_{WS_{2}}^{at-1.25V}$ > $I_{WSe_{2}}^{at-1.25V}$ . Here, we exclude single-layer MoSe_2_ due to its different junction area. This comparison is negatively correlated with the corrected Schottky barrier height for electrons from the SET study. The change in the current (the current difference between *V* = −0.25 V and *V* = −1.25 V at the low-resistive state at RESET) cannot be explained by thermionic emission. Nonetheless, image-force lowering and additional static lowering may also need to be considered as they are the specific characteristics of the reverse-biased Schottky diodes.^42^ We conclude that the current difference between *V* = −0.25 V and *V* = −1.25 V at the low-resistive state at RESET cannot be only explained using thermionic emission, image-force lowering, and additional static lowering due to Au dopants in the junction and the atomic thinness of the single-layer MoS_2_.

In the *V* = −0.25 V and *V* = −1.25 V range, it is clear that current deviates from the saturation and it does not follow a linear relation as shown in Figure 1(b). However, it also does not follow an exponential increase toward *V* = −1.25 V as it is shown in the representative *I*–*V* characteristics of the single-layer MoS_2_-based atomristor as shown in the atomristor article. These *I*–*V* characteristics might be related to the characteristics of atomically thin materials because band bending in atomically thin materials is limited due to weak dielectric screening.^43^ In this regard, we roughly read the current and voltage values at the low-resistive state at the RESET sweep from the atomristor article and the read values are given in Table S1 of the Supporting Information. Then, we plot the resistance, *R*_LRS_, versus electrical power, |*I*·*V*|, at −0.25 V, −0.5 V, −0.75 V, −1 V, and −1.25 V, as shown in Figure 4(a), which suggests a transport dominated by contact- or barrier-controlled carrier injection at low bias with progressive trap filling and defect-mediated trap-assisted tunneling at high bias. At low power, carrier injection occurs into empty trap states, corresponding to a relatively lower or moderate resistance (near Ohmic). As the bias increases toward −1.25 V, injected carriers occupy these traps and modify the local electrostatic potential and therefore modify the effective barrier at the interface. This trap filling leads to current increase slower than the voltage and therefore increase in resistance up to −0.75 V, as shown in Figure 4(a). When traps fill progressively, the system approaches a saturated configuration and leads to the observed saturation and slight downturn in resistance. At this point, we believe that the transport starts to transition. This behavior is consistent with contact-limited and trap-assisted transport under field. We further investigate the *d*(log­(*I*))/*d*(log­(|*V*|)) versus |*V*| relation, as shown in Figure 4(b). When *d*(log­(*I*))/*d*(log­(|*V*|)) is less than one as in the low biases up to −0.75 V, it indicates *I*–*V* behavior of contact- or barrier-limited injection at the following voltage biases: −0.25 V, −0.5 V, −0.75 V. When *d*(log­(*I*))/*d*(log­(|*V*|)) increases at high reverse bias, injection becomes easier due to field-assisted processes; such as effective barrier lowering or thinning and trap-assisted contribution.

**Figure 4:**
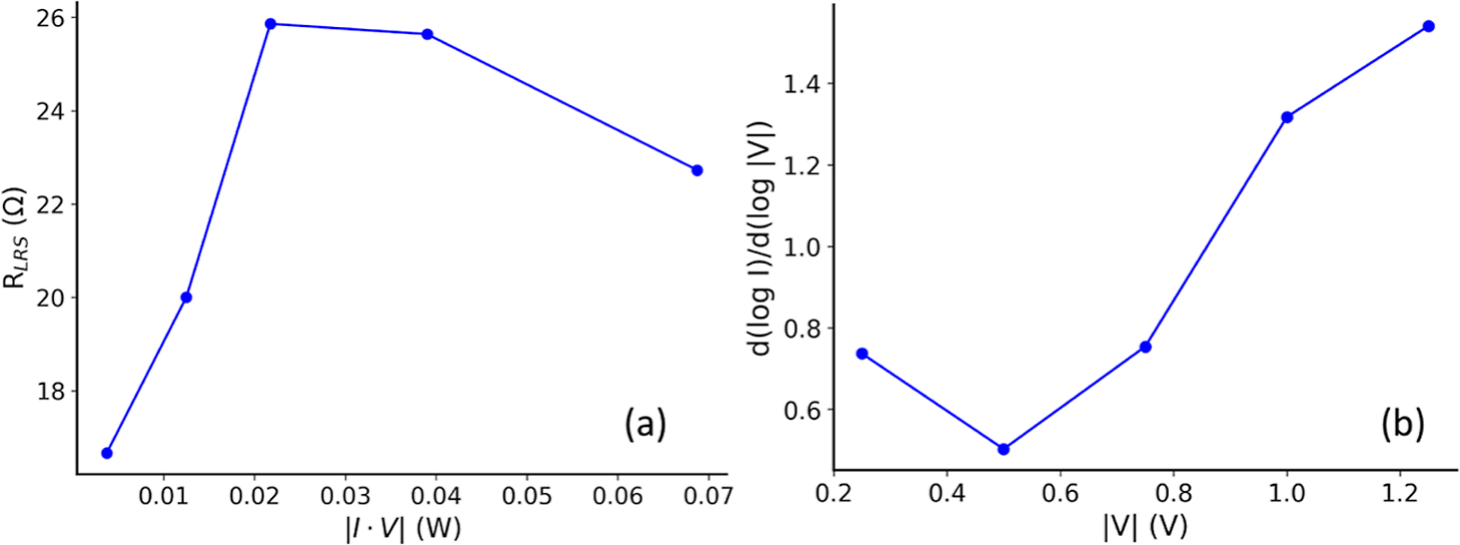
(a) Resistance at a low-resistive state, *R*_LRS_ = |*V*|/|*I*|, versus power, |*I*·*V*|, and (b) *d*(log *I*)/*d*(log |*V*|) versus |*V*|, where interior points inside the data set are calculated using central difference and the first and last points on the edges are calculated using forward and backward difference; less accurate. Current and voltage values are estimated visually from the atomristor’s *I*–*V* characteristics at −0.25 V, −0.5 V, −0.75 V, −1.0 V, and −1.25 V at the low-resistive state of the RESET sweep. Log represents the base-10 logarithm.

The *I*–*V* characteristics in region 1 in Figure 1(b) indicate the possibility of conduction with localized trap states due to Au dopants. It is reasonable that the traps are filled up to a certain level when we increase the reverse bias. Therefore, we need to look for the trap states and their effects in the single-layer MoS_2_-based atomristor. In this way, we present the electronic band structure of neutral Au-doped single-layer (3 × 3) MoS_2_ by replacing a sulfur with Au in a (3 × 3) single-layer MoS_2_ and we calculate the electronic band structure of the positively charged Au-doped (3 × 3) single-layer MoS_2_, which are shown in Figure 5(a),(b).

**Figure 5:**
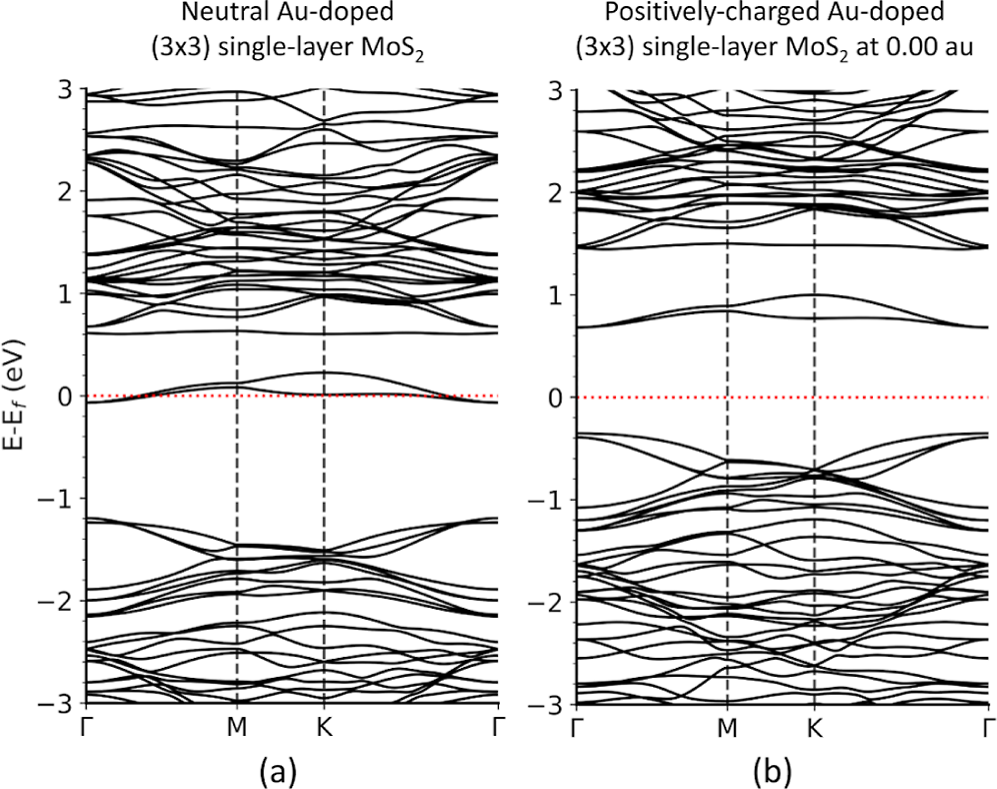
Electronic band structure of (a) neutral Au-doped (3 × 3) single-layer MoS_2_ and (b) positively charged Au-doped (3 × 3) single-layer MoS_2_ at an electric field amplitude of 0.00 au, where 0.00 au indicates the dipole correction. The electric field amplitudes are given in atomic units (au). The Fermi level is denoted by *E*_f_. The band structure is plotted along the high-symmetry path Γ-M-K-Γ. Panel (a) is adapted and redrawn from *ACS Appl. Electron. Mater*. 2025, **7**, 9, 3795–3809 (ref 12) under the Creative Commons Attribution 4.0 International License (CC-BY 4.0). Copyright Ⓒ 2025 The Authors. Published by the American Chemical Society.

We stick to a (3 × 3) supercell of single-layer MoS_2_ by fully acknowledging that the defect concentration in our DFT model differs by approximately an order of magnitude from experimentally averaged values. However, previous studies have shown that a (3 × 3) supercell for single-layer MoS_2_ indicates that artificial interactions between periodic images (spurious defect–defect interactions) are minimal for the quantities of interest.^44^ Also, the other reason to choose the (3 × 3) supercell is we aim to capture the essential local physics of defect-induced states in single-layer MoS_2_ rather than the global defect density of the entire flake. We have additionally carried out simulations with a larger (5 × 5) supercell using an atomic orbital-based Siesta approach with Siesta optimized structures (only atomic positions). The resulting band structures are presented in Figures S4 and S5 of the Supporting Information with slab dipole correction and without slab dipole correction. We confirm that the “split-off” state persists even at lower defect concentrations. We observed dopant-related in-gap DFT bands located almost in the DFT band gap in Figure 5(a),(b). Dopant-related DFT bands are close to the conduction band edge compared to the valence band edge in the supercell-based DFT band structure, the energy difference between the dopant-related DFT bands and the conduction band edge is around 0.6 eV at the Γ point in Figure 5(b), and we consider that these DFT bands might be important for electrical conduction.

In addition to in-gap DFT bands possibly acting as trap states, the metal and n-type semiconductor junction depletion region (depletion layer is formed in the semiconductor as defined in bulk semiconductor modeling) widens under reverse bias; therefore, the region without free charge carriers also widens, but there will be trap states. These trap states may lead to trap-assisted tunneling, and the current deviates slightly from saturation as discussed. For example, donor trap states are positively charged if they are empty (above the Fermi level), and trapped charges may also accumulate near the active electrode (top Au electrode) region.^33^ As it is discussed above, as one of the factors contributing to the current is the charges injected from the Schottky barrier, we can see that the corrected Schottky barrier height for electrons from the SET study negatively correlates with the current at the low-resistive state of the RESET as can be expected from the thermionic emission process. This means that increasing the effective Schottky barrier height for electrons leads to a low current. The junction with a high Schottky barrier height for electrons also has the lowest $\left|V_{RESET}\right|$ as read from the atomristor article; therefore, it is expected to have RESET early; namely, early and efficient filling of the trap states with increasing effective Schottky barrier height.

The increasing effective Schottky barrier height phenomenon for RESET is also suggested to explain the RESET operation in the atomristor article.^2^ Therefore, we want to test our prediction with the representative *I*–*V* characteristics presented in the atomristor article. We compare RESET voltages (based on the lowest absolute value of the voltage, where current first starts to drop significantly) in absolute values based on our reading from the representative *I*–*V* characteristics presented in the atomristor article as $\left|V_{RESET,MoS_{2}}\right|>\left|V_{RESET,WS_{2}}\right|>\left|V_{RESET,WSe_{2}}\right|$ , which is the inverse of the corrected Schottky barrier height comparison for electrons, ϕ_b,e_^*^, as given in the SET study, $\phi_{b,e,WSe_{2}}^{*}>\phi_{b,e,WS_{2}}^{*}>\phi_{b,e,MoS_{2}}^{*}$ , excluding the single-layer MoSe_2_-based atomristor because of its different junction area. Even though this comparison between the Schottky barrier height for electrons and the RESET voltage value aligns well based on the representative *I*–*V* characteristics in the atomristor article, we need to also consider the number of Au dopants absorbed into single-layer TMDCs in each cycle, as well as the trap energy levels. To conclude, effective Schottky barrier height is a function of defect density for single-layer MoS_2_ as it is discussed in the literature and defect density also determines the defect energy levels.^44^ Therefore, this comparison concludes that it is only valid for the materials with similar vacancy densities and therefore similar defect energy levels at a certain temperature, and at a certain junction area, like single-layer TMDCs with chalcogenide vacancies.

### RESET Process

We predict the mode of transport in the atomristor at the RESET sweep using eq 1. However, this was a rough approximation, and we also need to consider the band bending and energy-level alignments due to trap states and their dynamics. Therefore, the tunneling regime is valid only at low biases. We begin to study RESET by first carrying out a literature review of the *I*–*V* characterization studies conducted on single-layer MoS_2_-based atomristors. Based on our literature study, we classify *I*–*V* characteristics of atomristor into two distinct classes at the RESET for the single-layer MoS_2_-based atomristor, and these are tilted and abrupt. The tilted negative slope region is similar to a negative differential resistance, NDR, like behavior, which means that the *I*–*V* characteristics of region 2 depicted in Figure 1(b) is tilted and it has a gentler slope than the abrupt negative slope region. This means that the current decreases even though the voltage bias increases. We marked the current at the beginning of the RESET as *I*_peak_ and at the end of the RESET as *I*_valley_ in Figure 1(b) in accordance with NDR devices, even though we detect only a negative slope region.

To explain the decrease in current during RESET, we consider the so-called tilted negative slope region and abrupt negative slope regions. We interpret these NDR regions as a continuation of the trap dynamics and their characteristics observed in the junction just before region 2 in Figure 1(b), but as discussed by Leon Chua, a negative slope region implies a “phase lag” between the current and the voltage.^45^ This means that, the phase lag suggests a time-dependent process; namely, when we have an abrupt change, it suggests a faster device response. This means for tilted ones a process still continues in the negative slope region such as trap filling and therefore trap-assisted transport is suggested to continue in region 2 in Figure 1(b). In *I*–*V* characterization, an abrupt response can be induced while decreasing the carrier lifetime of trapped charges and therefore traps can be emptied easily and more efficiently and the charge carrier relaxation rate increases. For example, at high temperatures, charge carriers may relax or recombine more efficiently, and the lifetime of trapped charges may be shorter.^46^ We consider that for the tilted cases, not all the traps respond to the field in the semiconductor abruptly. The gradual activation or deactivation of traps results in a tilted negative slope region. Nonetheless, this characteristic of the negative slope region can change from cycle to cycle as well because in each cycle, the amount of Au in the semiconductor may vary; therefore, trap energy levels may change. For example, a transition from a tilted to an abrupt negative slope region is also observed with changes in Cl concentration in RbPbI_3–*x*_Cl_*x*_-based resistive switching devices in the literature; specifically, increased Cl concentration leads to an abrupt NDR.^47^ This discussion and its interpretation will allow us to further investigate the electronic structure of these junctions and look for the underlying reasons for RESET based on trap dynamics and their characteristics. For example, trap re-emission process is slower than the electron capture and during backward sweep, traps may still be partially filled.

Our literature study shows that the RESET and the negative slope region are related; therefore, we begin to look for a localized state (as the cause of the negative slope region) in the electronic structure of the Au-doped single-layer MoS_2_ through its energy-band diagrams and band alignments with the Au electrode. Nevertheless, investigating the electronic structure of the junction and the energy level alignments between the Au electrode and the Au-doped single-layer MoS_2_ together with their relative arrangements is not a straightforward procedure; especially when we consider asymmetric Au doping, its charge state, and the applied electric field. Nonetheless, we also see the importance of the time-dependent processes and therefore we start to understand RESET by identifying a localized state in the positively charged Au-doped (3 × 3) single-layer MoS_2_ under an electric field. This energy level must be different from those located near the middle of the DFT band gap, as shown in Figure 5(b). Therefore, we compute the electronic band structure of positively charged Au-doped (3 × 3) single-layer MoS_2_ at −0.02 au, −0.01 au, 0.01 au, and 0.02 au, as shown in Figure 6(a)–(d). We observe a localized state as a DFT band that is split from the conduction band at the Γ point, marked with a red arrow in Figure 6(a). Then, to compare neutral Au-doped (3 × 3) single-layer MoS_2_, positively charged Au-doped (3 × 3) single-layer MoS_2_ at 0.00 au, and positively charged Au-doped (3 × 3) single-layer MoS_2_ at −0.02 au, we enlarge their DFT bands around the Γ point as shown in Figure 7(a)–(c). Here, DFT bands are marked with a red arrow as given at 1.16 eV in Figure 7(c). Another approach to model Au-doped single-layer MoS_2_ might be to take a neutral Au-doped single-layer MoS_2_ at −0.02 au instead of positively charged Au-doped single-layer MoS_2_ at −0.02 au. In this way, we present the PDOS of neutral Au-doped single-layer MoS_2_ at −0.02 au in Figure S2 of the Supporting Information. Nevertheless, DFT bands of neutral Au-doped single-layer MoS_2_ at −0.02 au and positively charged Au-doped single-layer MoS_2_ at −0.02 au are expected to be similar. Therefore, we stick to positively charged Au-doped single-layer MoS_2_ at −0.02 au, even though Au dopants are neutralized under bias, because we may still need to consider positive charges due to lack of efficient injection.

**Figure 6:**
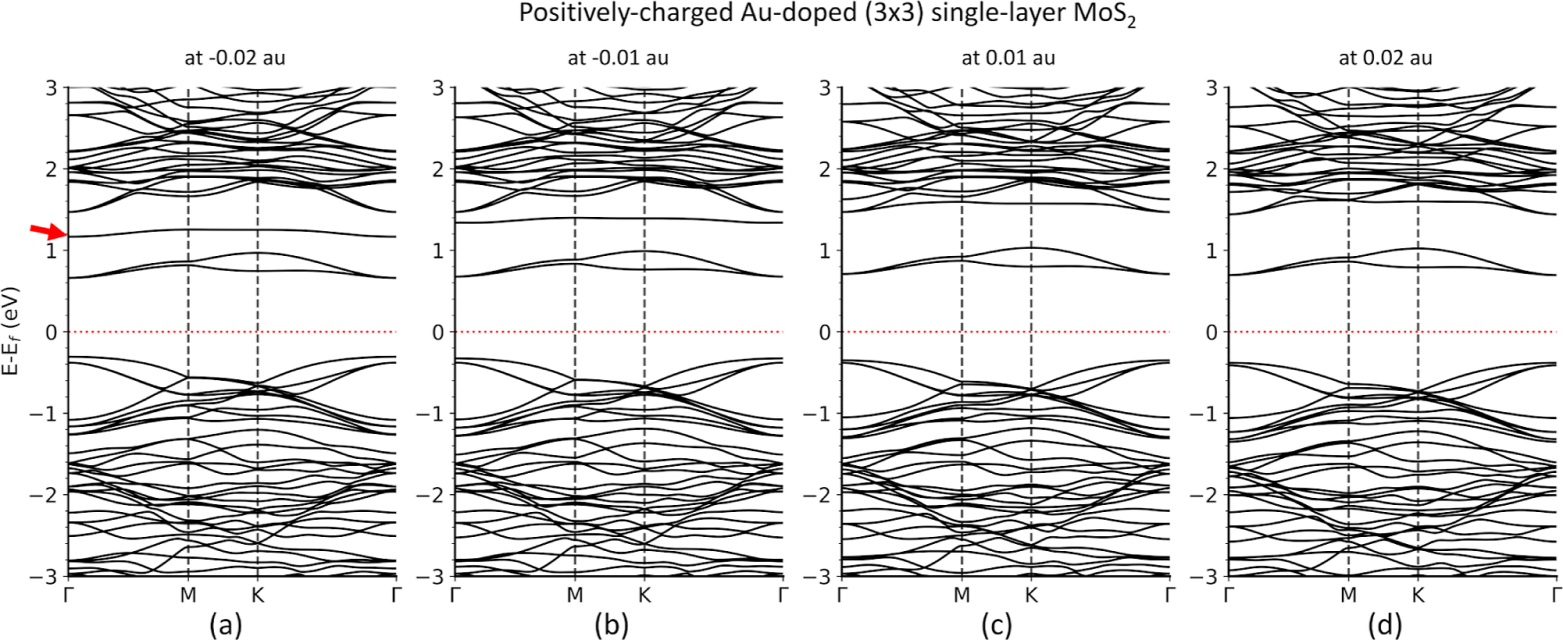
Electronic band structure of positively charged Au-doped (3 × 3) single-layer MoS_2_ at electric field amplitudes and directions of (a) −0.02 au, (b) −0.01 au, (c) 0.01 au, and (d) 0.02 au, where – represents the direction opposite of + in the *z*-axis. The electric field amplitudes are given in atomic units (au). The Fermi level is denoted by *E*_f_. The band structure is plotted along the high-symmetry path Γ-M-K-Γ. The red arrow in panel (a) indicates the split DFT band.

**Figure 7:**
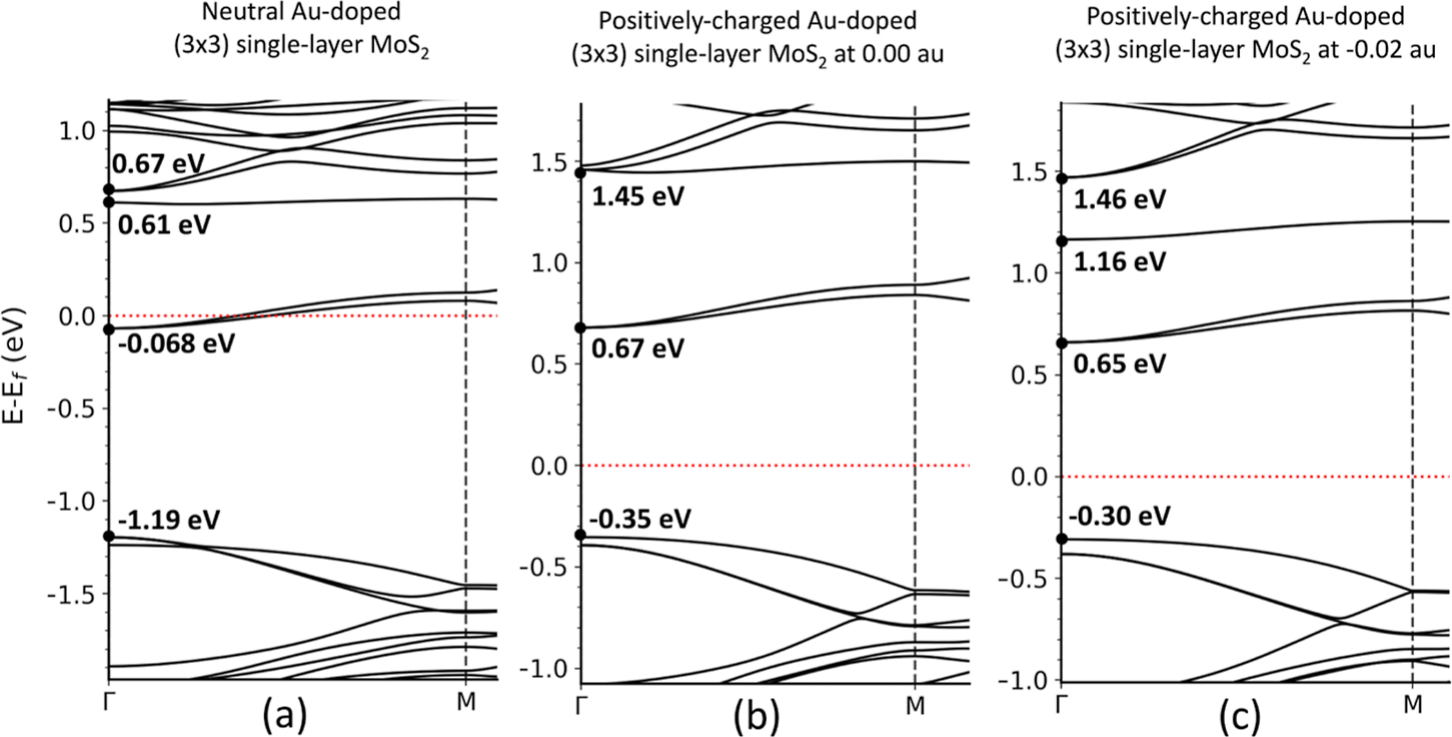
Enlarged electronic band structure around the Γ point of (a) neutral Au-doped (3 × 3) single-layer MoS_2_, (b) positively charged Au-doped (3 × 3) single-layer MoS_2_ at 0.00 au, and (c) positively charged Au-doped (3 × 3) single-layer MoS_2_ at −0.02 au, where 0.00 au indicates the dipole correction and – represents the direction opposite of + in the *z*-axis. The electric field amplitudes are given in atomic units (au). The Fermi level is denoted by *E*_f_. The band structure is plotted along the high-symmetry path Γ-M. Panel (a) is adapted and redrawn from *ACS Appl. Electron. Mater*. 2025, **7**, 9, 3795–3809 (ref 12) under the Creative Commons Attribution 4.0 International License (CC-BY 4.0). Copyright Ⓒ 2025 The Authors. Published by the American Chemical Society.

We investigate the split-off in-gap DFT band (red-marked DFT band) at different values of electric field and we understand that this DFT band split-off from the conduction band under applied electric field, where we study external electric field values up to −0.02 au, which approximately corresponds to −1 V/Å. Since we are using a supercell approach in the out-of-plane direction to model two-dimensional materials in our plane-wave-based DFT code, a spurious vacuum charging (charge spill out into the vacuum) may occur with high electric field values.^48,49^ However, we believe that any spurious charge leakage associated with the supercell approach is minute or negligible in our case. For this reason, we present the planar-averaged electrostatic potential energy profile of the positively charged Au-doped (3 × 3) single-layer MoS_2_ at 0.00 au in Figure S6a of the Supporting Information and at −0.02 au in Figure S6b of the Supporting Information. In both cases, Fermi level does not cut the electrostatic potential energy profile. We also visualize |ψ|^2^ through yellow isosurfaces, where ψ is the selected wave function, representing the red-marked DFT band in Figure 11, at the Γ point, as shown in Figure 8. To further check this, we carry out band structure calculations with electric field in an atomic orbital basis set-based and plane-wave-based DFT code of Siesta. These results are presented in Figure S3 of the Supporting Information, which shows split-off in-gap DFT bands similar to the ones obtained in Figure 7(c). We also visualize the wave function belonging to this DFT band at the Γ point indicates a localized wave function under −0.02 au; therefore, this state can trap charge carriers. The so-called split-off in-gap DFT band is due to electric field, namely, due to an electronic perturbation. Therefore, we can calculate the electronic perturbation potential, Δ*V*_elec_, as defined in the literature as the difference of Hartree contribution to total energy from the self-consistent field calculations as Δ*V*_elec_ = *V*_–0.02au_ – *V*_0.00au_, where *V*_–0.02au_ is the Hartree contribution to total energy from the self-consistent field calculations for positively charged Au-doped (3 × 3) single-layer MoS_2_ at −0.02 au and *V*_0.00au_ is the Hartree contribution to total energy from the self-consistent field calculations for positively charged Au-doped (3 × 3) single-layer MoS_2_ at 0.00 au.^41^ We found *V*_elec_ as it is equal to 2.2 eV. We should note that the electronic perturbation is responsible for the split-off in-gap DFT band. In addition to electric field, we also study electrostatic positive charging per cell in periodic boundary conditions with a plane-wave basis set. Since positive charging of the cell has no charge spill in contrast to negative charging case as discussed in the literature; therefore, we did not consider charge spilling due to electrostatic charging of the supercell in our case.^48,50^

**Figure 8:**
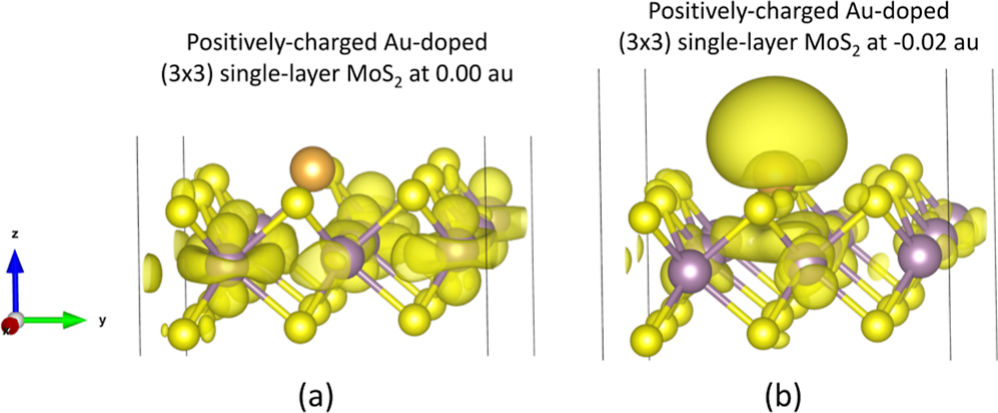
Visualization of |ψ|^2^, through yellow isosurfaces, where ψ is the selected wave function, representing the red-marked DFT band in Figure 11, at the Γ point for positively charged Au-doped (3 × 3) single-layer MoS_2_ at (a) 0.00 au and (b) −0.02 au, where 0.00 au indicates the dipole correction and – represents the direction opposite of + in the *z*-axis. The electric field amplitudes are given in atomic units (au). Bonds are represented as sticks, purple balls are Mo, yellow balls are S, and the orange ball is Au. Isosurface level of 0.001 (atomic units) is used.

We generate PDOS versus *E* – *E*_f_ relation for the Mo’s d orbital, Mo-d, S’s p orbital, S-p, Au’s s orbital, Au-s, and Au’s p orbital, Au-p, for the positively charged Au-doped (3 × 3) single-layer MoS_2_ at 0.00 au, as presented in Figure 9(a), and for the positively charged Au-doped (3 × 3) single-layer MoS_2_ at −0.02 au, as presented in Figure 9(b). We investigate the contribution of Au-dopant states to this split-off in-gap DFT band from Figure 9(b), where the main contributor to the split-off in-gap DFT band is Au-s after Mo-d. Therefore, the split-off in-gap DFT band is appeared to be a mixed state of different orbitals: these are mainly, Mo-d, Au-s, and S-p in this pseudopotential-based DFT study. Therefore, this DFT band is related to substitutional impurity atom with electronic perturbation. However, this standalone DFT band does not explain the atomristor’s mechanism in detail. Therefore, we need to generate its relation to the Au electrode by following the procedure given below.

**Figure 9:**
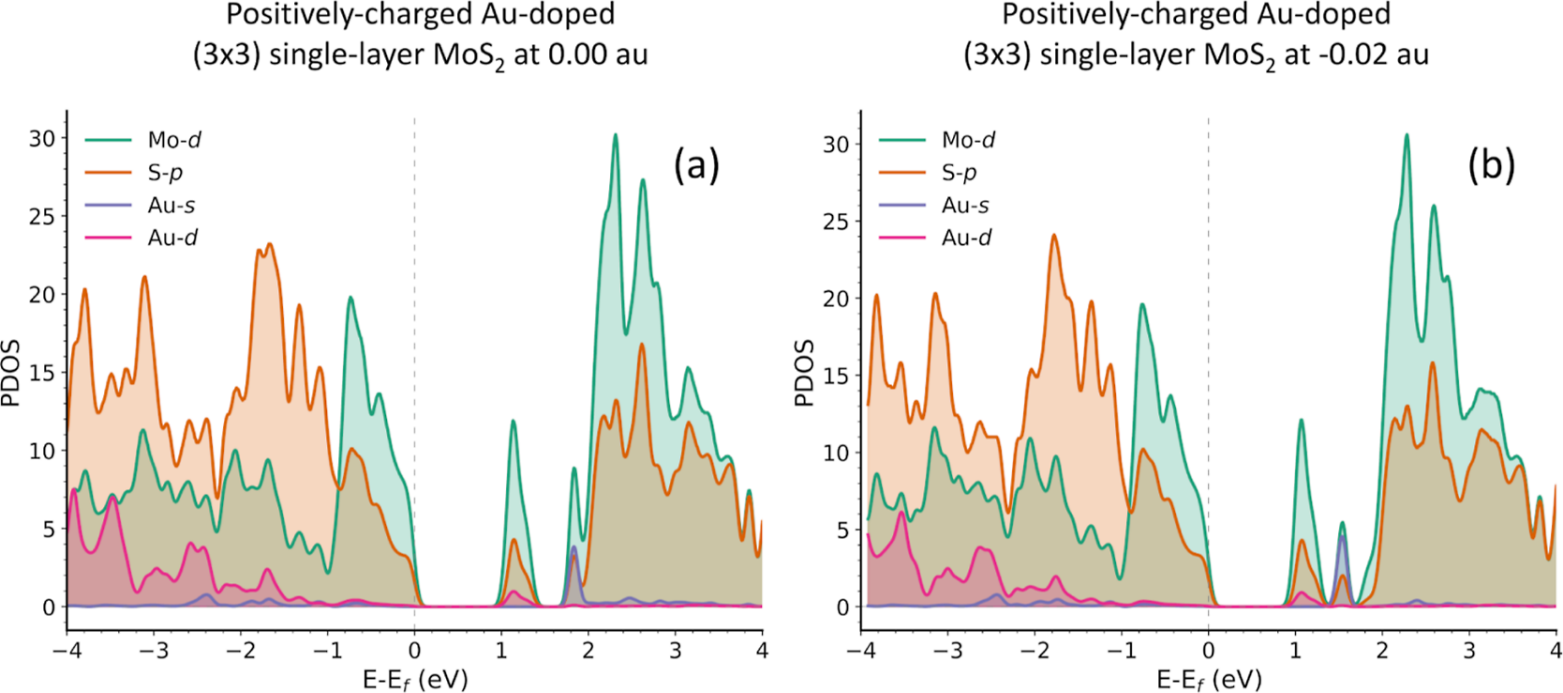
Projected density of states (PDOS) [eV/states/cell] for Mo’s d-orbital, Mo-d, S’s p-orbital, S-p, Au’s s-orbital, Au-s, and Au’s d-orbital, Au-d, versus *E* – *E*_f_ relation for positively charged Au-doped (3 × 3) single-layer MoS_2_ (a) at 0.00 au and (b) at −0.02 au, where 0.00 au indicates the dipole correction and – represents the direction opposite of + in the *z*-axis. The electric field amplitudes are given in atomic units (au). The Fermi level is denoted by *E*_f_. Plots are filled for visual reference.

Before explaining the procedure, we need to focus on the electronic structure of the Au electrode and then on the alignment of DFT bands around the Fermi level relative to the Au-doped single-layer MoS_2_. First, we obtain the work function of the Au electrode as 5.28 eV from the Supporting Information of the SET study. Then, the procedure follows: we calculate the interface potential step, Δ*V*_*i*_, at the interface between the Au electrode and the Au-doped (3 × 3) single-layer MoS_2_, based on an electrostatic potential energy plot as shown in the SET study. To calculate Δ*V*_*i*_, we take the slope of the electrostatic potential energy versus distance in the *z*-direction from the SET study, which is 0.017 eV/Å. Then, we consider the following equation to calculate the interface potential step, $\Delta V_{i}\approx \left(0.95\cdot L-d\right)\cdot E$ , where *L* is the supercell length in the *z*-direction and equals 32.87 Å, *d* is the thickness of the Au electrode and Au-doped single-layer MoS_2_ stack in the supercell and equals 13.57 Å, and E equals 0.017 eV/Å. We find Δ*V*_*i*_ approximately equal to 0.30 eV. This value will enable us to locate the vacuum levels of each material in the junction relative to one another, as shown in Figure 11(a), because 0.30 eV is the difference between vacuum levels of the Au electrode and the neutral Au-doped (3 × 3) single-layer MoS_2_.

After finding the vacuum-level differences between the Au electrode and the neutral Au-doped (3 × 3) single-layer MoS_2_ as 0.3 eV, we read the Fermi level of the Au-doped (3 × 3) single-layer MoS_2_ relative to the vacuum level from Figure 10(b), where we read it as 4.75 and 4.29 eV for the left-hand and right-hand side vacuum levels, respectively. Even though the Fermi level is arbitrary defined, this will enable us to locate the conduction band minimum with respect to the vacuum level. Then, we draw energy band diagrams based on DFT bands at the Γ point by considering the Au electrode and semiconductor as freestanding at the interface under a flat-band configuration with vacuum level offsets (without considering band bending), as shown in Figure 11(a),(b), where we consider the left vacuum level and the right vacuum level. For Figure 11(a),(b), we consider the electronic band structure of the neutral Au-doped (3 × 3) single-layer MoS_2_ as given in Figure 7(a). We find that the Fermi level of the Au electrode lies in between the conduction band edge and valence band edge of the neutral Au-doped (3 × 3) single-layer MoS_2_, where the conduction band edge is at 0.67 eV and valence band edge is at −1.19 eV as marked in Figure 7(a). We consider the DFT bands at the Γ point within the DFT band gap and mark them as red and green lines in Figure 11(a),(b). These energy levels are located at 0.61 eV and −0.068 eV at the Γ point, as given in Figure 7(a), and they might be attributed to the dopant and dopant-related effects.

**Figure 10:**
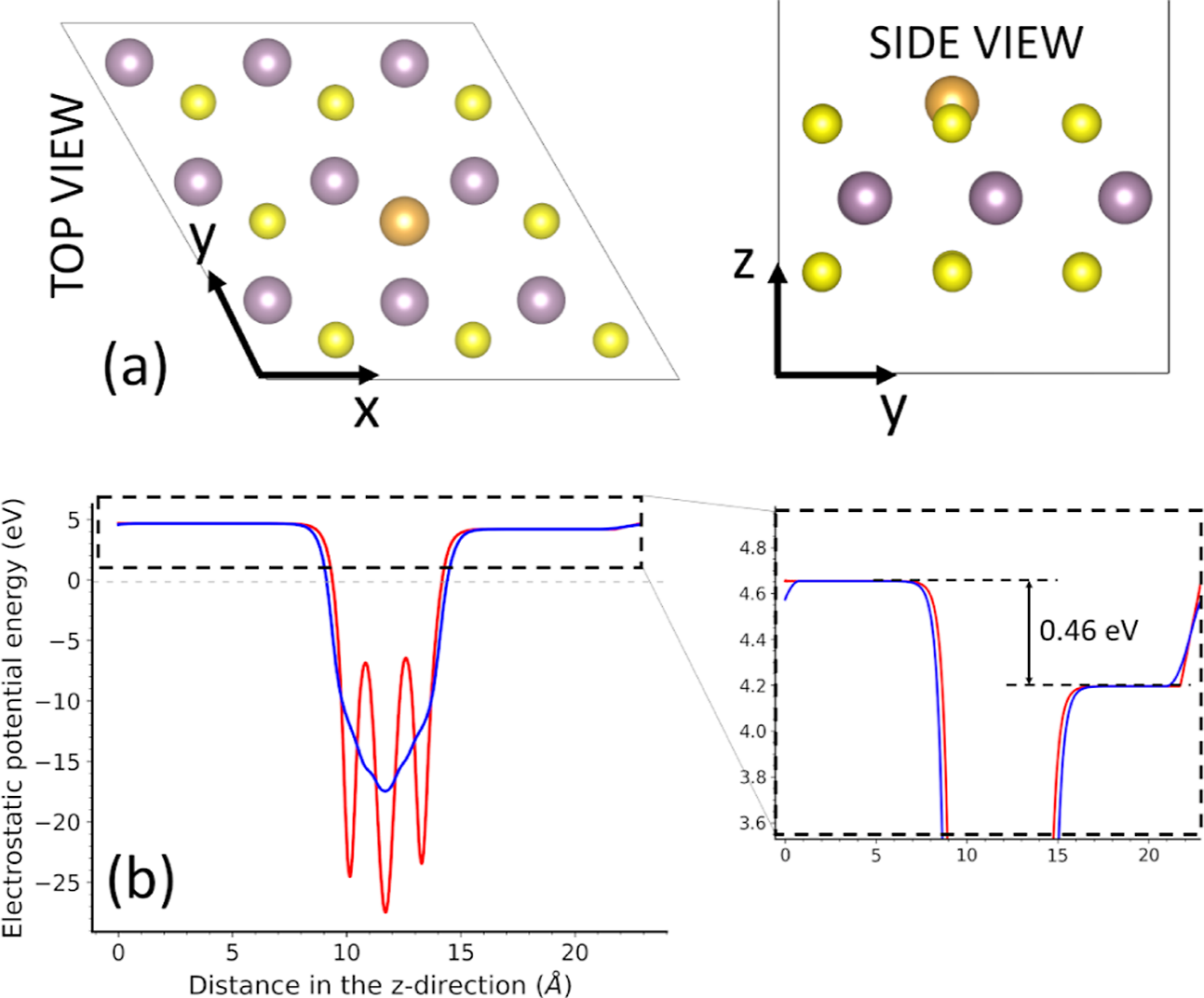
(a) Atomic structure of a Au-doped (3 × 3) single-layer MoS_2_ with its top view and side view, where purple balls are Mo, yellow balls are S, and the orange ball is Au and (b) electrostatic potential energy profile of Au-doped (3 × 3) single-layer MoS_2_ with dipole correction, with the inset showing its enlarged views.

**Figure 11:**
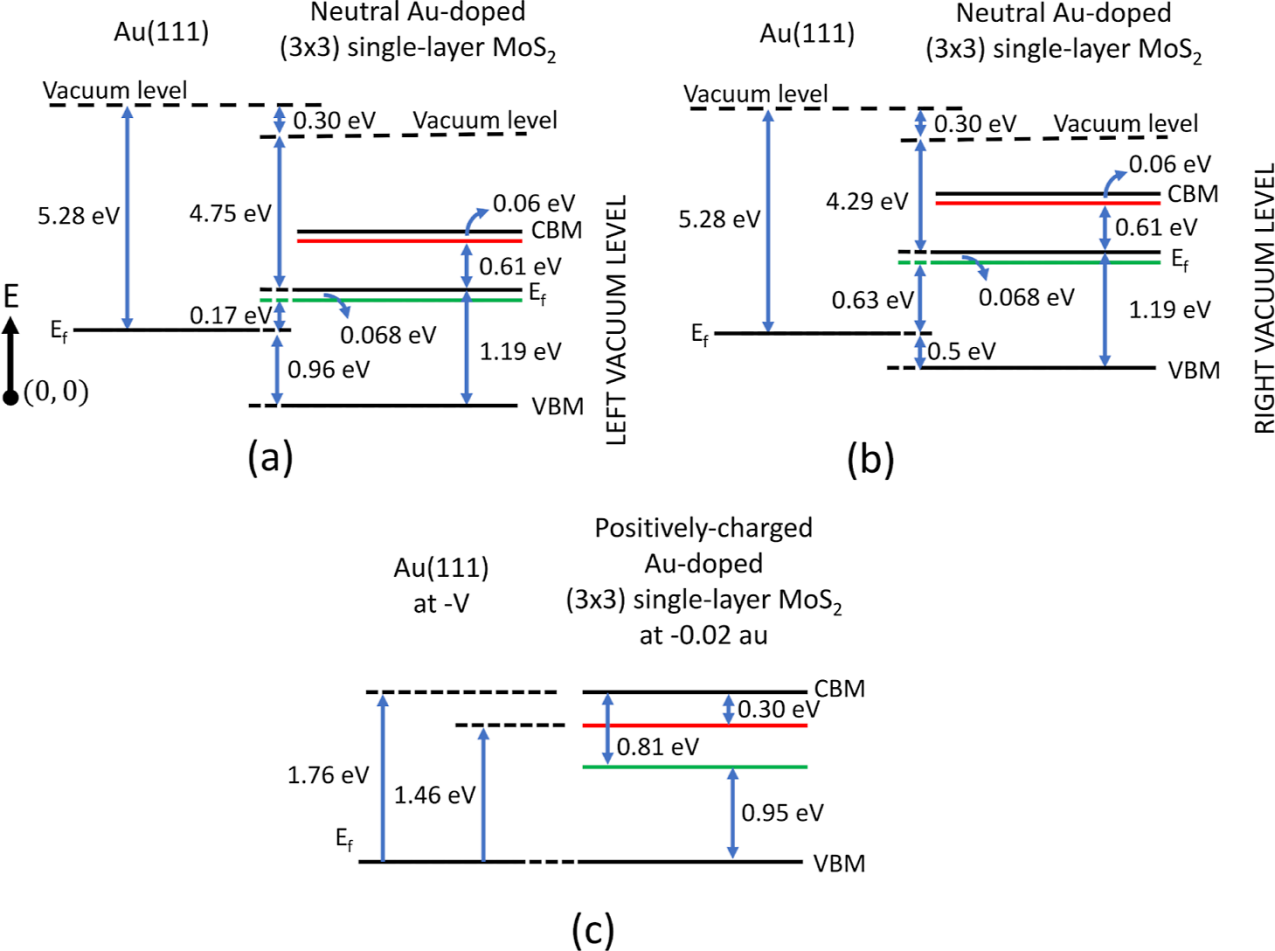
Schematic flat energy-band diagram (with vacuum level offset and without band bending) of a Schottky junction formed with a metal and a semiconductor for the junctions: Au(111)-neutral Au-doped (3 × 3) single-layer MoS_2_ by considering (a) the left vacuum level, (b) the right vacuum level, and (c) Au(111)-positively charged Au-doped (3 × 3) single-layer MoS_2_ at −0.02 au, where 0.00 au indicates the dipole correction and – represents the direction opposite of + in the *z*-axis. The electric field amplitudes are given in atomic units (au). The Fermi level is denoted by *E*_f_, valence band maximum is denoted by VBM, and conduction band minimum is denoted by CBM. Work function of the Au(111) electrode and energy differences between the DFT bands at the Γ point are marked with blue arrows, and they are approximate. Red and green lines are the DFT bands attributed to the effects of defects.

In Figure 11(a),(b), we present the energy bands of the Au electrode and the neutral Au-doped (3 × 3) single-layer MoS_2_’s and estimate their alignment based on their DFT bands at the Γ point. Nevertheless, we start to apply negative bias to the metal electrode and we have donor defect’s positive charge state, which is filling. If we first consider the case with a positive charge in the Au-doped (3 × 3) single-layer MoS_2_, we cannot precisely determine the interface potential step from DFT due to the overall bending of the electrostatic potential energy profile. Moreover, we know that the effect of charges requires a self-consistent solution of Poisson’s equation, accounting for interface dipoles and band bending. In addition to this, we also need to correct this energy by considering that this decharging does not occur instantly, because the decharging of the Au dopant occurs under bias, which is applied gradually in the atomristor article. Therefore, accurate determination is difficult in this DFT methodology. Nevertheless, we consider that the accurate energy levels are not essential in this comparative study; only the proportionality might be sufficient to discuss more about the abrupt current drop at *V*_RESET_.

In Figure 11(c), we locate the thermal equilibrium Fermi level of the device as aligned with the metal’s Fermi level and the semiconductor’s valence band maximum. We depict this configuration by considering the split-off in-gap DFT band of positively charged Au-doped (3 × 3) single-layer MoS_2_ at −0.02 au in Figure 11(c). Finally, we investigate the effect of this split-off in-gap DFT band using partial charge densities in real space for three different energy ranges from Fermi level. These are 1, 1.25, and 1.5 eV above the Fermi level for positively charged Au-doped (3 × 3) single-layer MoS_2_ at 0.00 au in Figure 12(a) and for positively charged Au-doped (3 × 3) single-layer MoS_2_ at −0.02 au in Figure 12(b). We saw that the Au dopant’s charge density in real space 1.5 eV above the Fermi level for positively charged Au-doped (3 × 3) single-layer MoS_2_ at 0.00 au is similar to Au dopant’s charge density in real space 1 eV above the Fermi level for positively charged Au-doped (3 × 3) single-layer MoS_2_ at −0.02 au.

**Figure 12:**
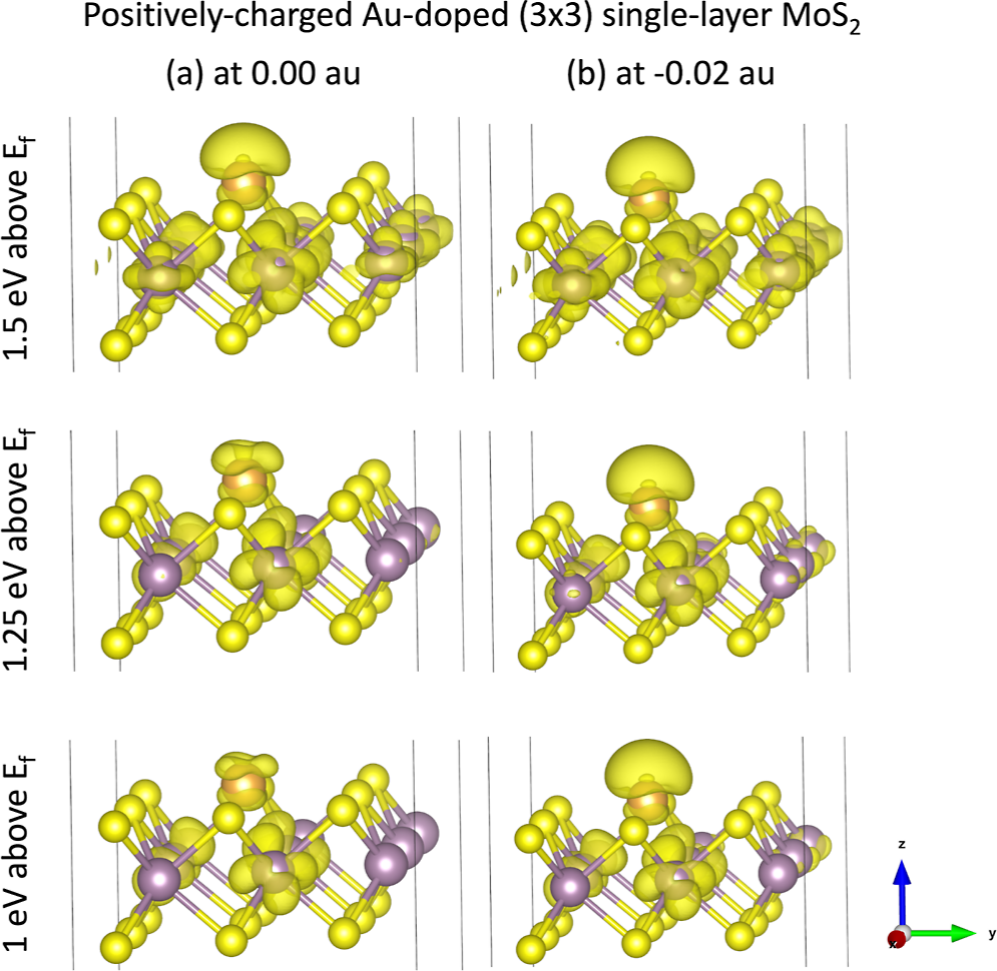
Visualization of partial charge density for a certain energy range in real space for DFT bands 1.5, 1.25, and 1 eV above *E*_f_ for positively charged Au-doped (3 × 3) single-layer MoS_2_ (a) at 0.00 au and (b) at −0.02 au, where 0.00 au indicates the dipole correction and – represents the direction opposite of + in the *z*-axis. The electric field amplitudes are given in atomic units (au). The Fermi level is denoted by *E*_f_. Bonds are represented as sticks, purple balls are Mo, yellow balls are S, and the orange ball is Au. Isosurface level of 0.009 (atomic units) is used.

In case of an applied voltage bias, we need to consider both metal electrodes; namely, the sandwiched structure of metal-resistive switching medium-metal structure (we neglect the Cr), because we need to shift the electrochemical potential of the contacts relative to each other. Since both electrodes are Au, we can neglect the built-in potential in our model. Then, we can depict the applied bias voltage as the arrows shifting the metal’s Fermi level upward with respect to the equilibrium Fermi level and we predict metal electrode’s Fermi level (although the varying quantity is electrochemical potential of the electrodes, we used the term metal electrode’s Fermi level for simplicity throughout this article) alignment with the green-marked DFT band, red-marked DFT band, and conduction band minimum at the Γ point as given in Figure 11(c). We indeed consider an upward shift in the Fermi level of the Au electrode when a negative voltage bias is applied to it. If this shift in the Fermi level is 1.46 eV, it can align with the red-marked energy level as given in Figure 11(c). Similarly, if this shift in the Fermi level is 1.76 eV, it can align with the conduction band minimum as given in Figure 11(c). These two values, 1.46 and 1.76 eV, represent the limits when we consider the thermal equilibrium Fermi level as in Figure 11(c). We note that the exact energy values are not important at a certain precision range due to our neglect of nonidealities in the Schottky junction, but the ratio (1.46 eV/1.76 eV) is important; it equals to 0.8, which also matches the (1.25 V/1.5 V) ratio, if we consider one significant digit (0.8), where 1.25 V is *V*_RESET_ and 1.5 V is *V*_max_, where *V*_RESET_ and *V*_max_ are shown in Figure 1(b), and values 1.25 and 1.5 V are taken from the atomristor article.

In this approach, it is important to consider energy levels separately because defect related energy levels may change based on the Au dopant concentration (we consider that the changes in Au concentration might be a reason why the *I*–*V* characteristics vary from cycle to cycle). Moreover, we set the energy difference between the Fermi level of the Au electrode and the valence band maximum of the semiconductor is 0.5 eV before biasing by considering the right vacuum level picture as shown in Figure 11(b). Also, energy levels might be changed due to possible spurious vacuum charge with the supercell approach in high electric fields as discussed. However, in the electronic band structure shown in Figure 6(b), we also observe the so-called split red DFT band at −0.01 au. Although its energy is shifted, the spurious vacuum charge at −0.01 au is expected to be nearly zero, indicating that this splitting is not driven by vacuum charge effects together with the results belonging to numerical atomic orbital-based calculations, as presented in Figure S3 of the Supporting Information. For all of these reasons, we first decide to investigate the ratio of (1.46/1.76) by writing it as Λ_ξ_ = (1.46 – ξ)/(1.76 – ξ), where ξ represents the shift in defect-related energy levels. Then, we evaluate Λ_ξ_ for ξ equal to 0, 0.1, 0.2, 0.3, 0.4, and 0.5 eV. We find that Λ_0_ = 0.82, Λ_0.10_ = 0.81, Λ_0.20_ = 0.80 and Λ_0.30_ = 0.79, Λ_0.40_ = 0.77, and Λ_0.50_ = 0.76. These ratios are very close to each other and to 0.82. For example, we calculate the percent error between 0.76 eV (from Λ_0.50_) and 0.83 eV (from 1.25 V/1.5 V) as |(0.76−0.83)/0.83×100%≈8%| . Therefore, our proportionality-based approach may be useful to explain the RESET of the atomristor. Finally, we also calculate Λ_0_ for the −0.01 au case, as shown in Figure 6(b) and Figure S3a,b of the Supporting Information, where we also have a split red DFT band. We found Λ_ξ_ at ξ = 0 as 1.66/1.79 = 0.92 from Figure 6b, 1.43/1.72 = 0.83 from Figure S3a of the Supporting Information, and 1.55/1.80 = 0.86 from Figure S3b of the Supporting Information.

The ratio established by *V*_RESET_ over *V*_max_, (1.25 V/1.5 V) = 0.83, as read from the atomristor article is close to Λ_ξ_ for ξ equal to 0, 0.1, 0.2, 0.3, 0.4, and 0.5 eV. This may indicate that the choice of −0.02 au in our DFT studies may correspond to the experimental applied voltage bias value in the atomristor article. We discuss this correspondence further. For this, we first write |−0.02| au as equal to 1.02 × 10^10^ V·m^–1^ based on the conversion provided by Quantum ESPRESSO as given in the Computational Methods section. Similarly, based on our calculations in Section 4 of the Supporting Information and as presented in Figure S8 of the Supporting Information, we predict the average electric field in the junction at −1.2 V as (1/2) $\left|E_{\max}\right|$ , which is equal to 2.07 × 10^9^ V·m^–1^ for N_d_^2‑D^ = 10^14^ cm^–2^, but this value is highly affected from defect concentration and it is based on the bulk model; therefore, we can also calculate the maximum electric field using 1.25 *V*/0.7 × 10^–9^ m ≈ 2 × 10^9^ V·m^–1^. We found the electric field value of 2.07 × 10^9^ V·m^–1^ from analytical calculations, which will be considered as the effective experimental electric field. To conclude, 1.02 × 10^10^ V·m^–1^ is obtained from DFT studies, which is one or 2 orders of magnitude larger than the experimental one as it is also stated in the literature^51,52^ Therefore, the choice of −0.02 au accurately reflects the experimental conditions of the single-layer MoS_2_-based atomristor.

As stated by Herbert Kroemer, “the interface is the device”; therefore, we must also consider the Au electrode to explain the abrupt decrease in current at RESET, which constitutes an interface with single-layer MoS_2_.^53^ We provide a multiscale interpretation rather than a complete interface-resolved description, although we acknowledge the importance of explicit interface calculations to connect atomistic modeling and device-level behavior. For this, we first consider the electronic band structure of the positively charged Au-doped single-layer MoS_2_ under an electric field of −0.02 au, as represented in Figure 7(c), in addition to the neutral and positively charged Au-doped single-layer MoS_2_ electronic band structures. Then, we establish the interface band configuration with the Au electrode, as shown in Figure 11(c). In this picture, when we shift the Fermi level of the Au electrode upward relative to the valence band maximum of the semiconductor in the junction, we first reach the green energy level, as depicted in Figure 11(c). Then, electrons from the Au electrode may tunnel into this level when the metal’s Fermi level aligns with the trap energy level. When electrons occupy the green energy level, the dopants may become neutralized, since dopants located above the Fermi level are positively charged.

When we continue to increase the applied voltage bias, the Fermi level of the metal electrode aligns with the red-marked energy level in Figure 11(c). Electrons may tunnel to this red energy level and neutralize some of the positive charges. However, this is highly dependent on the voltage sweep rate. As it is given in the literature, we can mark the green level as the deep trap level and the red level as the shallow donor level with respect to the green DFT band or defect-related state, which can trap charge, and we also know that all shallow donors will be ionized above the Fermi level and only deep impurities near the surface and above the Fermi level will ionize and deep donor level impurities; namely, green energy level can only respond the slow signals.^36,54^ Therefore, depending on the sweep rate, the red level might become neutralized, while the green level may retain some positively charged dopants. When the red level is neutralized, electrons coming will either be excited to the conduction band or will relax into the green energy level, as shown in Figure 11(c) under bias. Electrons trapped in defect states can be excited to the conduction band when the temperature is high under an electric field, and this contribution is known as the Poole–Frenkel effect, and it is investigated in Section 5 of the Supporting Information, and the barrier lowering versus electric field relation is depicted in Figure S9 of the Supporting Information.^55^ Even though the electric field can effectively fit for the Poole–Frenkel effect, we neglect the Poole–Frenkel effect in our model, and we set the rate-limiting step in our transport model as related to traps and the Schottky barrier.

When electrons in the red energy level relax to the green energy level, they may neutralize the dopants associated with the green energy level in the semiconductor. Therefore, this relaxation occurs at an energy level difference of (1.16 eV–0.65 eV) based on Figure 7(c). On the other hand, in the absence of an applied electric field, the energy level difference is (1.45 eV-0.67 eV) based on Figure 7(b). Even though these energy level differences are lower than the energy differences related to holes for trap-assisted recombination, which are (0.65 eV–(−0.30 eV)) and (0.67 eV–(−0.35 eV)), we consider these electron-related energy differences because these two energy levels are electron traps and therefore relaxation times are usually longer (slower) due to Coulomb repulsion, which lowers the capture probability, as it is also discussed in the literature.^56^ On the other hand, cross-section can be increased and relaxation times might be shorter due to high electron–phonon coupling. Therefore, we expect that the energy levels are related to electron traps: (1.16 eV–0.65 eV) and (1.45 eV–0.67 eV); therefore, charge carrier relaxation will dominate in this comparative study, and recombination with holes in the valence band may not become the determinant mechanism to set the rates.^57^ Thus, trap state’s neutralization is a determinant of the RESET. Because the relaxation proceeds in nanoseconds, it may not be resolved in a direct current sweep. What the direct current sweep records is the subsequent stable resistance state, in which the metal Fermi level aligns with the red DFT band and moves toward the conduction band minimum. To conclude, the RESET process is centered around Au-induced localized states, which can act as efficient recombination or charge exchange centers without forming a dense trap band. We also consider the contribution of oxygen dopants (substitute S vacancy) in single-layer MoS_2_ based on a literature work since the measurements are taken under ambient conditions in the atomristor article; we detect that band structure with in-gap states induced by the Au dopant presented in this article is not akin to the oxygen-doped band structure of single-layer MoS_2_.^58^

The so-called trap-to-trap transition rate may be estimated from the energy difference of (1.16 eV–0.65 eV), which equals 0.51 eV. Nevertheless, this rate may also be valid when the Fermi level shifts just above the red energy level due to excitations. Therefore, a definitive determination may not be possible. This may help explain the cycle-to-cycle variations in the RESET behavior of single-layer MoS_2_-based atomristor *I*–*V* characteristics. On the other hand, as shown in Figure 7(b), in the absence of an electric field, the transition in the semiconductor is determined by the energy difference of (1.45 eV–0.67 eV), which is equal to 0.78 eV. These energy differences will allow us to predict changes in current based on the capture rates, $R_{\Delta E}$ , defined as $R_{\Delta E}\propto \exp\left(-\Delta E/\left(kT\right)\right)$ , where Δ*E* is taken as the energy difference between DFT bands at the Γ point.^59^ Namely, when we have a small Δ*E*, we can have a higher capture rate. This higher capture rate may enable the filling of trap states in a certain voltage sweep window.

We calculate the transition rates as a ratio, $R_{\Delta E_{1}}/R_{\Delta E_{2}}$ , of above-mentioned Δ*E*s, where Δ*E*_1_ is 0.51 eV as read from Figure 7c and Δ*E*_2_ is 0.78 eV as read from Figure 7(b), that is $R_{0.51eV}/R_{0.78eV}$ = 3.2 × 10^4^, where we consider $R_{\Delta E}\propto \exp\left(-\Delta E/\left(kT\right)\right)$ at *T* ≈ 300 K. The ratio of 3.2 × 10^4^ is close to the *I*_peak_/*I*_valley_ ratio, which is approximately 6 × 10^4^, as calculated from the representative *I*–*V* characteristics presented in the atomristor article, where *I*_peak_ is equal to 5.5 × 10^–2^ A and *I*_valley_ is equal to 9 × 10^–7^ A based on our approximate reading from the atomristor article. These ratios 3.2 × 10^4^ from $R_{0.51eV}/R_{0.78eV}$ and 6 × 10^4^ from *I*_peak_/*I*_valley_ do not match exactly. Nevertheless, thermal excitation should also be considered when calculating the $R_{\Delta E_{1}}/R_{\Delta E_{2}}$ ratio. We estimate the effect of this excitation by calculating the following two ratios: $R_{\left(\Delta E_{1}+kT\right)}/R_{\Delta E_{2}}$ and $R_{\left(\Delta E_{1}-kT\right)}/R_{\Delta E_{2}}$ , which are calculated to be 1.1 × 10^4^ and 8.7 × 10^4^, respectively. We conclude that 1.1 × 10^4^ < (*I*_peak_/*I*_valley_) < 8.7 × 10^4^, where (*I*_peak_/*I*_valley_) = 6 × 10^4^. Therefore, this approach may explain the current drop during RESET. Moreover, this transition may heat the device during its operation due to its nonradiative nature. To conclude, we estimate the trap-to-trap relaxation process in this comparative study through ratios based on energy differences read from the DFT bands at the Γ point.

To calculate the $R_{\Delta E_{1}}/R_{\Delta E_{2}}$ ratio, we consider the numerical values from the charged supercell calculations with a finite electric field in a plane wave DFT code. Nevertheless, there are two concerns in this approach. The first one is mimicking the experimental conditions: this charge state should be stable during RESET in a contacted device; therefore, this positively charged Au defect should be understood as a quasi-stable state. Hence, we carry out only electric field calculations in an atomic orbital-based DFT code of Siesta, and the split-off DFT bands are also apparent in the band structure, as shown in Figure S4 of the Supporting Information. The second concern is about the computational methods because the charge with its background-charge compensation is not suggested to be used with the dipole correction in DFT. Therefore, we also carry out calculations without dipole slab correction, as shown in Figure S5 of the Supporting Information, and the split-off DFT bands are still apparent in the band structure, although the dipole correction is suggested to be used with finite electric field simulations in DFT.

We state the importance of the red and green DFT bands as shown in Figure 11(c); therefore, we decide to model Au-doped single-layer MoS_2_ as a two-level system. By doing this, we can study the population dynamics of these energy levels under external perturbation. This study will provide ways for the future research to further improve the device performance for the enhanced endurance performance, reduced power consumption, and better mimicry of human synaptic dynamics. Here, we focus on synaptic dynamics as an example: The population difference between the red and green energy levels oscillates over time under external perturbation in resonance with Δ*E* and oscillation amplitude decreases if we have a finite phase relaxation rate, as shown in Figure S10 of Section 6 in the Supporting Information. On the other hand, if the phase relaxation rate is zero, we can observe a constant amplitude of oscillations over time. Therefore, if we consider a finite phase relaxation rate for the red energy level, the time evolution of the population difference may mimic synaptic depression if the atomristor is perturbed by an external source at *V*_RESET_ in resonance with Δ*E*. For this analysis, we solve the Maxwell–Bloch equations as described in Section 6 of the Supporting Information.^60,61^

Fermi-level alignment with the semiconductor energy levels is of utmost importance for *V*_RESET_, but its exact determination is challenging. However, clearly defining this boundary is challenging due to excitations, such as thermal excitations. In addition to thermal excitations, we need to also explain the discrepancy between the exact energy levels in our DFT study and those observed experimentally. This difference in energy levels might be due to nonidealities such as strain, pinholes, and cracks in atomically thin materials or at the Schottky barrier interface.^62^ Nonetheless, a proportionality-based approach may remain valid even without considering the effects of nonidealities, but still some nonidealities may evolve over time during device operation, and these dynamic effects are not captured in our so-called proportionality-based approach through ratios. We mark the dopant-related energy levels relative to the conduction band edge as 0.3 and 0.81 eV for the red and green energy levels, respectively, as shown in Figure 11(c). Quantitatively, the same energy-level differences are also reported in the literature as 0.33 eV as the barrier for Fowler–Nordheim tunneling and 0.82 eV for trap-assisted tunneling in an experimental MoS_2_-based atomristor study.^63^ There are beyond-DFT approaches (including spin–orbit coupling) for more accurate energy-level determinations, but the so-called proportionality-based approach might be enough to explain the atomristor’s operation principle. Precise determination of energy levels may not be necessary, because we may assume that when the Fermi level of the metal electrode is slightly above the red energy level, the transition rate may begin to be determined by the energy difference (1.16 eV–0.65 eV), and *V*_RESET_ might occur before the metal’s Fermi level aligns with the 1.46 eV energy level due to thermal excitations as given in Figure 7(c). Moreover, defect concentration can change during operation and these dopant-related energy levels can change, as a result.

Charge carrier–phonon coupling in single-layer MoS_2_ as given in the literature may lower the energy of certain phonon modes in single-layer MoS_2_, especially the acoustic modes around the high-symmetry point M in the Brillouin zone, a phenomenon known as phonon softening.^64,65^ As shown in the literature, phonon softening may result in abrupt negative differential resistance due to quadratic electron–phonon coupling.^66^ Further discussion of this speculation on electron–phonon coupling in RESET is beyond the scope of this article. However, it is also clear that the material may not withstand the excess doping at some point and therefore some structural transitions might be possibly observable. This kind of increased charge carrier and phonon coupling might be a good indication of thermally induced lattice distortions due to local heating; namely, doping, may create states, which can act as charge traps as it is discussed in the literature.^67^ This additional state may facilitate the charge carrier relaxation or nonradiative recombination and therefore may increase heat in the junction and therefore may further induce structural distortions. Finally, talking about structural changes in the structure after the Au dopant, we need to consider the Au-doped single-layer MoS_2_ in Figure 10, where the difference in distance in the *z*-direction between the Au dopant and the upper plane of sulfur is 0.43 Å for the neutral Au case and 0.47 Å for the positively charged Au case after relaxation with fixed lattice cell parameters. These differences may indicate the distortion of bonds. In addition to this, recent literature also claims about the structural changes (a phase transition from the 2H phase to 1T′ phase) in the single layer after Au adsorption to vacancies in the single layer.^68^

As it is discussed above, the red-marked energy level in Figure 11(c), compared to Figure 11(b), might be important to explain the abrupt decrease in the current at *V*_RESET_ in the atomristor. We found that the splitting of the red-marked energy level from the conduction band edge at the Γ point under electric field is similar to the formation of vertical quantum-well states in few-layer MoS_2_ in the absence of an electric field but with an increasing number of single-layer MoS_2_ layers as shown in the literature.^69^ Nonetheless, the sharp drop in current is due to charge carrier relaxation possibly enhanced by quadratic electron–phonon coupling because impurities couple nonlinearly with lattice vibrations. At some point, charge carriers may relax or recombine via multiphonon emission instead of contributing to the current, leading to an abrupt negative slope region.^66^

Finally, we depict the negative crystal orbital Hamiltonian population, −COHP, with respect to *E* – E_f_ for Mo–Au and S–Au interactions in Figure 13(a) and in Figure 13(b) for neutral Au-doped single-layer MoS_2_ and positively charged Au-doped single-layer MoS_2_, where in the positively charged case, we only modify self-consistent field calculation script by adding, tot_charge = 1. In these plots, positive values on the *x*-axis represent the bonding characteristics and the negative values on the *x*-axis represent the antibonding characteristics. Similarly, positive values on the *y*-axis represent the unoccupied states and the negative values on the *y*-axis represent the occupied states. In Figure 13(a), strong bonding peaks are around *E* – *E*_f_ = −5 eV, strong antibonding peaks are around *E* – *E*_f_ = 0.65 eV, and we have some antibonding states around the Fermi level. Here, S–Au interactions have smaller magnitude compared to Mo–Au interactions. Therefore, we conclude that the Mo–Au interaction dominates the stability of the Au dopant and also we conclude that bond strengths are sensitive to charge or occupation changes due to presence of antibonding states near the Fermi level. For example, when we remove an electron from the system and calculate the COHP again, as shown in Figure 13(b), occupations and bonding contributions will change. Therefore, Figure 13(a),(b) shows a slightly decreased peak (less negative −COHP) for the Mo–Au interaction and a slightly increased peak (more negative −COHP) for the S–Au interactions at around *E* – *E*_f_ ≈ 1.3 eV. This indicates decreased antibonding characteristics (0.88 eV change in COHP) for Mo–Au interaction and increased antibonding characteristics (0.06 eV change in COHP) for S–Au interaction. Nevertheless, the blue peak at around *E* – *E*_f_ ≈ 1.3 eV in Figure 13(b) corresponds to a strong unoccupied Mo–Au antibonding state, which can weaken the Mo–Au bond if we inject electrons to the system. This local bond weakening may lead to possible structural changes. We conclude that the bonding characteristics are of utmost importance for understanding the RESET of the atomristor. Nonetheless, the last analysis using the LOBSTER code does not account for the Au electrode, but it provides insights into the charge transport with a possibility of bonding changes in RESET.

**Figure 13:**
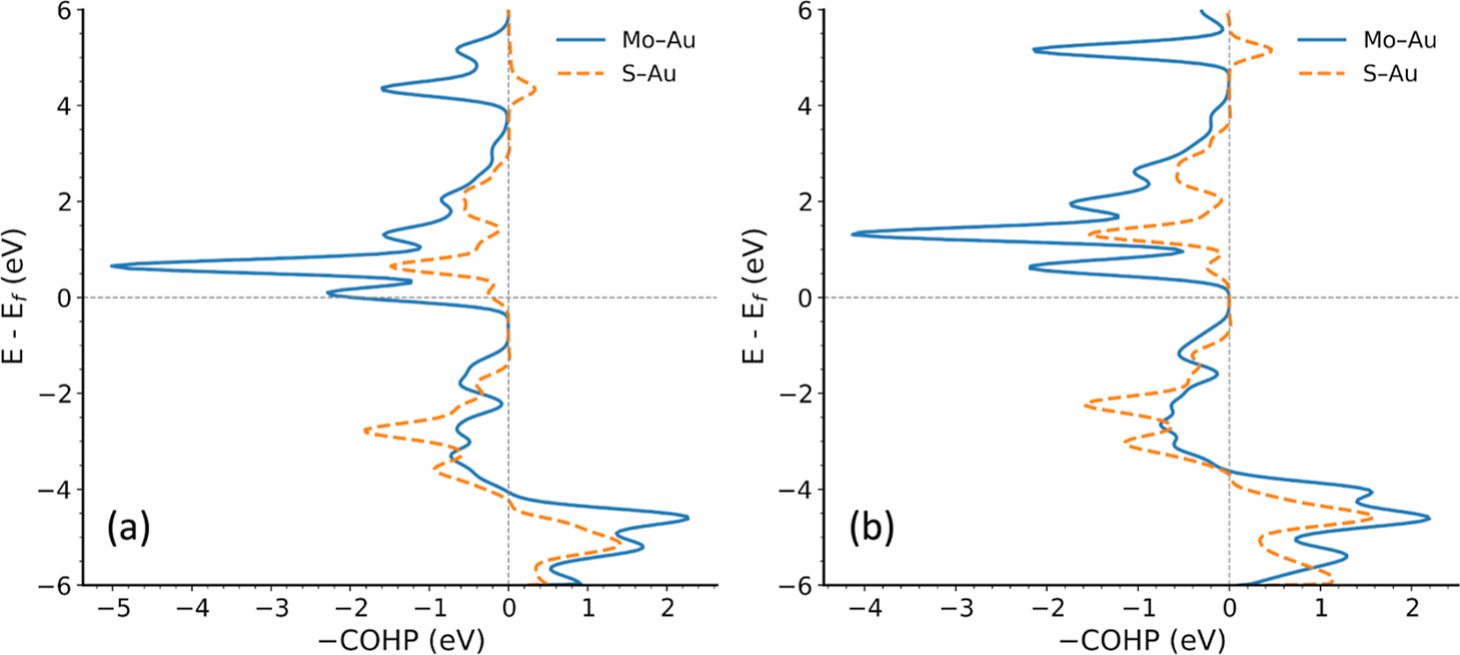
Energy, *E*, relative to the Fermi level, *E* – *E*_f_, versus negative crystal orbital Hamiltonian population, −COHP, relation for Mo–Au and S–Au interactions in (a) neutral Au-doped (3 × 3) single-layer MoS_2_ and for (b) positively charged Au-doped (3 × 3) single-layer MoS_2_. The represented contributions are summed based on the COHP values obtained from the three nearest Mo atoms surrounding the Au dopant in single-layer MoS_2_ for Mo–Au and the COHP values obtained from the six nearest S surrounding the Au dopant in single-layer MoS_2_.

### High-Resistive State after the RESET Process

At the end of region 2 in Figure 1(b) at the RESET voltage in the atomristor article, we observe the so-called current bump, which is a small rise in the current with an increase in voltage under negative polarity and then a fall in the current as marked by the blue dashed lines (region 3) in Figure 1(b). This tiny bump at the end of the RESET line is attributed to charge transport into the semiconductor when their energy levels align; namely, when the energy difference between the Fermi level of the Au electrode and the conduction band edge of the semiconductor is reduced in the flat-band picture and also due to tunneling of charge carriers through states inside the band gap. In the case of the single-layer MoS_2_-based atomristor, this current is expected to begin increasing after the Au electrode’s Fermi level aligns and surpasses the red marked energy level in Figure 11(c) and most probably increase significantly after 1.5 V based on the representative *I*–*V* characteristics presented in the atomristor article. This electron transport from the Au electrode to single-layer MoS_2_ may lead to the overheating or thermal damage. Therefore, experimental researchers limit the maximum voltage in the RESET sweep (*V*_max_) to enable multicycle device operation as shown in the atomristor’s *I*–*V* characteristics in region 3 in Figure 1(b). Although allowing electrons transport into the semiconductor is necessary to establish the hysteresis, it must be optimized because it also increases heat dissipation. Therefore, this is a critical stage for optimizing power dissipation.

After the current bump, we read the current values at the *V*_RESET_ for different TMDC-based atomristors from the atomristor article. We define this current as the valley current, *I*_valley_, and compare it as $I_{valley,MoS_{2}}>I_{valley,WSe_{2}}>I_{valley,WS_{2}}$ , based on the representative *I*–*V* characteristics presented in the atomristor article. In this analysis, we exclude the characteristics of single-layer MoSe_2_ due to its different junction size. We read the current values at the high-resistive state of the RESET from the *I*–*V* characteristics presented in the atomristor article at −0.5 V and compared them as $I_{MoS_{2}}^{at-0.5V}>I_{WSe_{2}}^{at-0.5V}>I_{WS_{2}}^{at-0.5V}$ . This corresponds to the *I*_valley_’s comparison above. Then, we compare the (*I*_peak_ – *I*_valley_) differences based on the representative *I*–*V* characteristics in the atomristor article as (*I*_peak_ – *I*_valley_)­WS2 > (*I*_peak_ – *I*_valley_)­WSe2 > (*I*_peak_ – *I*_valley_)­MoS_2_. We know that this (*I*_peak_ – *I*_valley_) can be linked to the charge carrier lifetime, τ. Therefore, we estimate the charge carrier lifetime in each TMDC by considering the defect formation diagrams from Figure S12 of the SET study, even though they are based on chalcogenide vacancies. We can estimate the chalcogenide vacancy defect formation energies as WS_2_ > WSe_2_ > MoS_2_ from the “SET study”. We assume that the lower the defect formation energy, the higher the trap densities; therefore, the lower the charge carrier lifetime. Since we assume a common switching mechanism across all TMDC junctions, we expect the following, $\tau_{WS_{2}}$ > $\tau_{WSe_{2}}$ > $\tau_{MoS_{2}}$ . Therefore, (*I*_peak_ – *I*_valley_) and τ positively correlate with each other assuming similar trap energy levels, capture cross sections, and trap concentrations in each TMDC-based junction.

When we release the voltage from *V*_RESET_ to 0, a barrier forms, and therefore, current will decrease. The increasing effective Schottky barrier is also an important parameter governing the *I*–*V* characteristics of the high-resistive state of the RESET process under the reduced negative polarity of the applied voltage bias. Compared to the Schottky barrier formation during SET in the high-resistive state, the high-resistive state in the RESET sweep shows a higher dependence on temperature than the high-resistive state in the SET sweep. This high-temperature dependence under negative voltage polarity is also evident in the atomristor article, where there is an asymmetry in the current values between negative and positive polarities of the applied bias with temperature. Therefore, this indicates an additional temperature-dependent process of trap-assisted tunneling contribution at high bias. In addition to this observation from the experimental data in the atomristor article, we observe an increasing $\left|V_{RESET}\right|$ value with an increasing sweep rate, as depicted in the atomristor article. Even though the sweep rates used are high, this dependence of the sweep rate on $\left|V_{RESET}\right|$ also correlates with trap filling and charge carrier relaxation processes during RESET. At high sweep rates, the response of the traps to the filling process can only reach a comparable response level at higher electric fields, namely, at high $\left|V_{RESET}\right|$ , compared to low sweep rates. However, this RESET is also affected by the number of operating cycles of the atomristor, the Au concentration, and the trap levels.

The high-resistive state at the RESET sweep indicates progressive trap defilling under decreasing bias. To investigate this, we first plot the log I versus d(log|V|) relation as shown in Figure 14(a) based on *I*–*V* values read from the atomristor article and the read values are also given in Table S2 of the Supporting Information. Then, we depict the d(log⁡I)/d(log|V|) versus |V| in Figure 14(b). At −1.25 V, the ratio of d(log⁡I)/d(log|V|) is more than three, this indicates a strong field-assisted transport. Therefore, any extra charge can contribute to current quickly as it is in the case of current bump discussed, while values with the ratio greater than 2 are the trap-mediated modulation of the effective injection barrier; such as trap-assisted tunneling. The progressive decrease in d(log⁡I)/d(log|V|) with decreasing bias voltage is consistent with field-induced modulation of the effective Schottky barrier and trap-assisted transport. This behavior reflects dynamic trap defilling and redistribution of the interfacial electrostatics during the RESET process. We also study the instantaneous power consumption as shown in Figure 14(c) and we conclude that when traps are filled at high voltages, we observe an increased instantaneous power consumption and when traps are emptied at low voltages, we observe low instantaneous power consumption. Additionally, nonlinear instantaneous power versus voltage may indicate a field-driven process and barrier-controlled mechanism rather than filamentary conduction. The overall mechanism can be summarized as a dynamic Schottky barrier controlled by trap occupancy.

**Figure 14:**
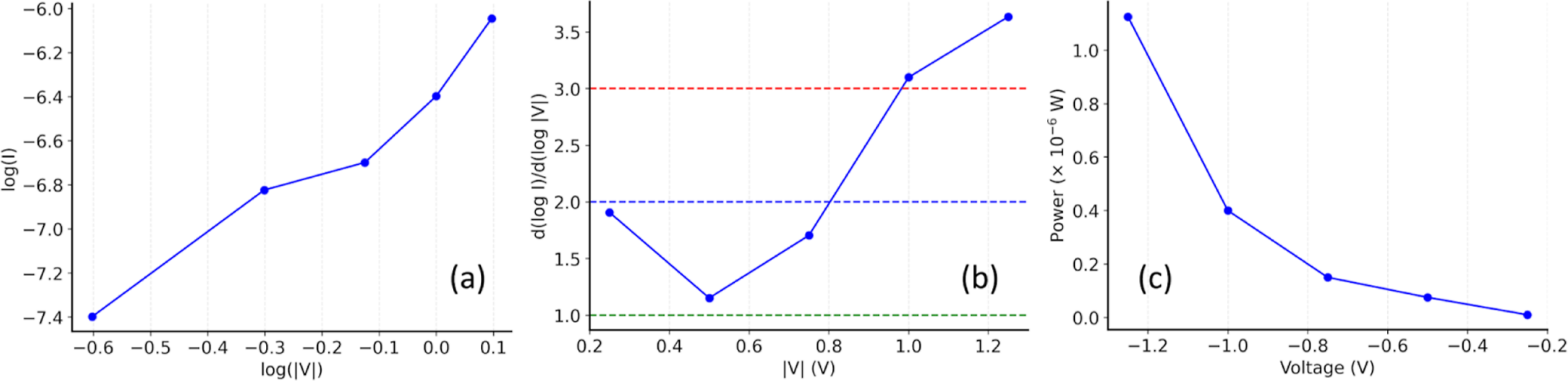
(a) Current, log­(*I*), versus voltage, (log|V|) , (b) d(log⁡I)/d(log|V|) versus |*V*|, where interior points inside the data set are calculated using central difference and the first and last points on the edges are calculated using forward and backward difference; less accurate, green, blue, and red horizontal lines are visual guides, and (c) power (calculated using |I−V| ) versus voltage relation. Current and voltage values are estimated visually from the atomristor’s *I*–*V* characteristics at −1.25 V, −1.0 V, −0.75 V, −0.5 V, and −0.25 V at the high-resistive state of the RESET sweep. Log represents the base-10 logarithm.

We consider that at −1.25 V, all donors are filled and therefore we have the highest current value at the high-resistive state at the RESET sweep. Then, toward −0.25 V, the Fermi level moves close to the valence band and number of ionized donors increase and current decreases because effective barrier height increases due to accumulation of positively charged donors at the interface. Here, accumulation of fixed charges at the interface changes the electrostatic potential strongly (∇^2^ϕ∝*N*_d_^+^) due to two-dimensional nature of single-layer MoS_2_, where we have weak screening; charges modify the potential strongly. Then, changing electrostatic potential leads to bands bending upward and therefore effective barrier height, Φ̅, increases and this reduces the carrier injection controlled current (∼exp(−Φ̅/kT)) . To conclude, the increasing *N*_d_^+^ toward 0 V at the high-resistive state of the RESET sweep indicates a decreasing depletion width or screening length in the junction. Therefore, Au dopants will be positioned close to the interface due to decreasing depletion layer (screening length). This is a similar conclusion as stated in the literature studies, which were talking about the incorporation of a Au atom back to the Au electrode.^70^ Therefore, even though the Au dopants are still at the interface over cycles of operation and affect the endurance performance, the junction is prepared for the new sweep cycle.

As stated by Bhattacharjee et al., large current and power density are required for the RESET of an atomristor.^63^ We consider the power consumed during the RESET as equal to the multiplication of *I*_peak_ and *V*_RESET_, where *I*_peak_ and *V*_RESET_ are depicted in Figure 1(b). Here, we first consider *I*_peak_ and how to decrease it to reduce the power consumption. However, decreasing *I*_peak_ to reduce power consumption may not be feasible if we want to achieve a high (ON/OFF) current ratio and maintain a large hysteresis window during the RESET sweep. We can also consider decreasing *V*_RESET_, which can be done by increasing the effective barrier height for the electrons. The latter is valid if the other factors, such as trap energy levels and density, are the same in a comparative manner. In addition to *I*_peak_ and *V*_RESET_, *I*_valley_ is also important for studying power consumption.

Throughout our analysis of the atomristor, we assume a uniform thickness of single-layer material over the junction area and we took the thickness as 7 Å based on its experimental thickness given in the atomristor article measured using atomic force microscopy. However, an accurate thickness can also be considered as the interlayer spacing of the bulk MoS_2_, which is 6.12 Å.^71^ Moreover, we mainly extract experimental data and interpret the experimental results from the atomristor article and from another article, where the atomristor’s switching properties are investigated.^63^ In the latter article, researchers used the chemical vapor deposition technique to fabricate MoS_2_ single layers with MoO_3_ seed films, and then the atomristor was fabricated using optical lithography. On the other hand, in the atomristor article, the metal–organic chemical vapor deposition technique was used with Mo­(CO)_6_ precursor gas, and then the atomristor was fabricated using electron-beam lithography. We did not consider the differences between the fabrication methods in our approach and we also assumed that the representative *I*–*V* characteristics presented in the literature are free from artifacts and experimental errors during our interpretation.

## Conclusion

We model and study the RESET sweep of the bipolar resistive switching in a single-layer MoS_2_-based atomistor. We test our claims using DFT based on a Au-doped single-layer MoS_2_ and Au electrode junction, and we compare the results with the experimental data presented mostly in the atomristor article. We depict the operation based on the most dominant charge transport type in the junction in our model, even though many modes can coexist and dynamically compete. We conclude that power consumption and endurance characteristics are related to variations in the number of Au dopants in the single-layer MoS_2_-based atomristor. Finally, our work provides instances of how to combine *I*–*V* characterization results with DFT in a collaborative manner together with analytic models. Nevertheless, there are nonidealities in Schottky junctions, especially with low-dimensional materials in experimental studies, which pose a modeling challenge. Therefore, we used the so-called proportionality establishment approach to overcome some of these challenges between computational and experimental studies in a comparative study.

In future studies, device-to-device and cycle-to-cycle variations, such as those caused by changes in Au dopant concentration, can be analyzed with statistical methods; such as average *I*–*V* characteristics rather than representative ones, to quantify the variations in atomristors together with the probabilistic and dynamic nature of these devices; such as simulating Au dopants together with sulfur vacancies and their time-dependent concentration changes is necessary for a correct interpretation of RESET. Even though we argue that electron transport is mainly determined by the top Au electrode and the single-layer MoS_2_, the thin-Cr layer’s reaction with environmental oxygen and its contribution to resistive switching together with capacitance might be investigated in the future. Moreover, we propose to study the dynamics of charge carriers in the single-layer MoS_2_-based atomristor through light control to achieve greater precision than voltage bias alone. Finally, the atomristor’s operation might be investigated at low sweep rates in the future. Today, understanding the working mechanism of these atomically thin devices relies on performing experiments such as *I*–*V* characterization and analyzing results with first-principle methods in a multiscale simulation scheme.

## Supplementary Material



## Notes

Multiscale modeling of the RESET sweep in a single-layer MoS_2_ atomristor using density functional theory: details of computational methods, current–voltage values for the RESET sweep, additional electronic structure calculations, bulk Schottky barrier model, Poole–Frenkel barrier lowering, and Maxwell–Bloch equations (PDF)

## References

[1] Ielmini, D. ; Waser, R. Resistive Switching: From Fundamentals of Nanoionic Redox Processes to Memristive Device Applications; John Wiley & Sons, 2015.

[2] Ge R., Wu X., Kim M., Shi J., Sonde S., Tao L., Zhang Y., Lee J. C., Akinwande D. (2018). Atomristor:
nonvolatile resistance switching in atomic sheets of transition metal
dichalcogenides. Nano Lett..

[3] Zhu K., Vescio G., González-Torres S., López-Vidrier J., Frieiro J. L., Pazos S., Jing X., Gao X., Wang S.-D., Ascorbe-Muruzábal J. (2023). Inkjet-printed
h-BN memristors for hardware security. Nanoscale.

[4] Lanza M., Sebastian A., Lu W. D., Le Gallo M., Chang M.-F., Akinwande D., Puglisi F. M., Alshareef H. N., Liu M., Roldan J. B. (2022). Memristive technologies for data storage, computation,
encryption, and radio-frequency communication. Science.

[5] Woo K. S., Williams R. S., Kumar S. (2025). Localized Conduction Channels in
Memristors. Chem. Rev..

[6] Lemme M. C., Akinwande D., Huyghebaert C., Stampfer C. (2022). 2D materials for future
heterogeneous electronics. Nat. Commun..

[7] Tian H., Deng B., Chin M. L., Yan X., Jiang H., Han S.-J., Sun V., Xia Q., Dubey M., Xia F. (2016). A dynamically reconfigurable
ambipolar black phosphorus
memory device. ACS Nano.

[8] Park T., Jeong H., Park S.-O., Hong S. M., Seo S., Park S., Choi S. (2023). The effect of Schottky barrier modulation
on conduction and failure mechanisms of an Ag/WOx/p-Si based memristor. J. Appl. Phys..

[9] Zhou H., Sorkin V., Chen S., Yu Z., Ang K.-W., Zhang Y.-W. (2023). Design-dependent switching mechanisms
of Schottky-barrier-modulated
memristors based on 2D semiconductor. Adv. Electron.
Mater..

[10] Nirmal K. A., Kumbhar D. D., Kesavan A. V., Dongale T. D., Kim T. G. (2024). Advancements
in 2D layered material memristors: unleashing their potential beyond
memory. npj 2D Mater. Appl..

[11] Khot S., Jung D., Kwon Y. (2023). Finite-element
simulation of interfacial
resistive switching by Schottky barrier height modulation. J. Comput. Electron..

[12] Turfanda A., Gagliardi A. (2025). Single-Layer MoS2-Based Atomristor’s Resistive
Switching Model for SET Sweep with Density Functional Theory Simulations. ACS Appl. Electron. Mater..

[13] Jain A., Ong S. P., Hautier G., Chen W., Richards W. D., Dacek S., Cholia S., Gunter D., Skinner D., Ceder G., Persson K. a. (2013). The Materials Project: A materials
genome approach to accelerating materials innovation. APL Mater..

[14] Giannozzi P., Baroni S., Bonini N., Calandra M., Car R., Cavazzoni C., Ceresoli D., Chiarotti G. L., Cococcioni M., Dabo I. (2009). QUANTUM ESPRESSO: a
modular and open-source software project for quantum simulations of
materials. J. Phys.: Condens. Matter.

[15] Giannozzi P., Andreussi O., Brumme T., Bunau O., Buongiorno
Nardelli M., Calandra M., Car R., Cavazzoni C., Ceresoli D., Cococcioni M. (2017). Advanced capabilities
for materials modelling with Quantum ESPRESSO. J. Phys.: Condens. Matter.

[16] Giannozzi P., Baseggio O., Bonfà P., Brunato D., Car R., Carnimeo I., Cavazzoni C., De Gironcoli S., Delugas P., Ferrari Ruffino F. (2020). Quantum ESPRESSO toward
the exascale. J. Chem. Phys..

[17] Monkhorst H. J., Pack J. D. (1976). Special
points for Brillouin-zone integrations. Phys.
Rev. B:.

[18] Grimme S., Antony J., Ehrlich S., Krieg H. (2010). A consistent
and accurate
ab initio parametrization of density functional dispersion correction
(DFT-D) for the 94 elements H-Pu. J. Chem. Phys..

[19] Grimme S., Ehrlich S., Goerigk L. (2011). Effect of the damping function in
dispersion corrected density functional theory. J. Comput. Chem..

[20] Perdew J. P., Burke K., Ernzerhof M. (1996). Generalized
gradient approximation
made simple. Phys. Rev. Lett..

[21] Schlipf M., Gygi F. (2015). Optimization algorithm for the generation
of ONCV pseudopotentials. Comput. Phys. Commun..

[22] Hamann D. (2013). Optimized
norm-conserving Vanderbilt pseudopotentials. Phys. Rev. B:Condens. Matter Mater. Phys..

[23] Van
Setten M. J., Giantomassi M., Bousquet E., Verstraete M. J., Hamann D. R., Gonze X., Rignanese G.-M. (2018). The PseudoDojo:
Training and grading a 85 element optimized norm-conserving pseudopotential
table. Comput. Phys. Commun..

[24] Freysoldt C., Neugebauer J. (2018). First-principles calculations for
charged defects at
surfaces, interfaces, and two-dimensional materials in the presence
of electric fields. Phys. Rev. B.

[25] Momma K., Izumi F. (2011). VESTA 3 for three-dimensional visualization
of crystal, volumetric
and morphology data. J. Appl. Crystallogr..

[26] Maintz S., Deringer V. L., Tchougréeff A.
L., Dronskowski R. (2016). LOBSTER: A
tool to extract chemical bonding from plane-wave based DFT. J. Comput. Chem..

[27] Deringer V.
L., Tchougréeff A. L., Dronskowski R. (2011). Crystal orbital
Hamilton population (COHP) analysis as projected from plane-wave basis
sets. J. Phys. Chem. A.

[28] Dal
Corso A. (2014). Pseudopotentials periodic table: From H to Pu. Comput. Mater. Sci..

[29] Soler J. M., Artacho E., Gale J. D., García A., Junquera J., Ordejón P., Sánchez-Portal D. (2002). The SIESTA
method for ab initio order-N materials simulation. J. Phys.: Condens. Matter.

[30] García A., Papior N., Akhtar A., Artacho E., Blum V., Bosoni E., Brandimarte P., Brandbyge M., Cerdá J. I., Corsetti F. (2020). Siesta:
Recent developments
and applications. J. Chem. Phys..

[31] Troullier N., Martins J. L. (1991). Efficient pseudopotentials for plane-wave calculations. Phys. Rev. B.

[32] Bengtsson L. (1999). Dipole correction
for surface supercell calculations. Phys. Rev.
B.

[33] Funck C., Menzel S. (2021). Comprehensive model of electron conduction
in oxide-based
memristive devices. ACS Appl. Electron. Mater..

[34] Yang J. J., Pickett M. D., Li X., Ohlberg D. A., Stewart D. R., Williams R. S. (2008). Memristive switching mechanism for
metal/oxide/metal
nanodevices. Nat. Nanotechnol..

[35] Suryavanshi S. V., Gabourie A. J., Barati Farimani A., Pop E. (2019). Thermal boundary conductance
of two-dimensional MoS2 interfaces. J. Appl.
Phys..

[36] Sze, S. M. ; Ng, K. K. Physics of Semiconductor Devices; John Wiley & Sons, Ltd, 2006; pp 134–196.

[37] Shi Y., Huang J.-K., Jin L., Hsu Y.-T., Yu S. F., Li L.-J., Yang H. Y. (2013). Selective
decoration of Au nanoparticles
on monolayer MoS2 single crystals. Sci. Rep..

[38] Sreeprasad T., Nguyen P., Kim N., Berry V. (2013). Controlled defect-guided,
metal-nanoparticle incorporation onto MoS2 via chemical and microwave
routes: electrical, thermal, and structural properties. Nano Lett..

[39] Liu H., Grasseschi D., Dodda A., Fujisawa K., Olson D., Kahn E., Zhang F., Zhang T., Lei Y., Branco R. B. N. (2020). Spontaneous chemical functionalization via
coordination of Au single atoms on monolayer MoS2. Sci. Adv..

[40] Rhoderick E. H. (1982). Metal-semiconductor
contacts. IEE Proc. Solid State Electron. Dev..

[41] Zunger A., Malyi O. I. (2021). Understanding doping of quantum materials. Chem. Rev..

[42] Woods N., Hall S. (1994). On the contribution
of recombination currents in Schottky barrier
diodes. Semicond. Sci. Technol..

[43] Raja A., Chaves A., Yu J., Arefe G., Hill H. M., Rigosi A. F., Berkelbach T. C., Nagler P., Schüller C., Korn T. (2017). Coulomb
engineering of the bandgap and excitons in
two-dimensional materials. Nat. Commun..

[44] Sorkin V., Zhou H., Yu Z. G., Ang K.-W., Zhang Y.-W. (2022). The effects
of point defect type, location, and density on the Schottky barrier
height of Au/MoS2 heterojunction: a first-principles study. Sci. Rep..

[45] Chua, L. Handbook of Memristor Networks; Springer, 2019; pp 197–230.

[46] Klein P., Myers-Ward R., Lew K.-K., VanMil B., Eddy C., Gaskill D., Shrivastava A., Sudarshan T. (2010). Recombination
processes controlling the carrier lifetime in n- 4H–SiC epilayers
with low Z1/2 concentrations. J. Appl. Phys..

[47] Das U., Das D., Paul B., Rabha T., Pattanayak S., Kanjilal A., Bhattacharjee S., Sarkar P., Roy A. (2020). Induced Vacancy-Assisted
Filamentary Resistive Switching Device Based on RbPbI3–x Cl
x Perovskite for RRAM Application. ACS Appl.
Mater. Interfaces.

[48] Gürel H. H., Özçelik V. O., Ciraci S. (2013). Effects of charging
and perpendicular electric field on the properties of silicene and
germanene. J. Phys.: Condens. Matter.

[49] Wang X.-P., Li X.-B., Chen N.-K., Zhao J.-H., Chen Q.-D., Sun H.-B. (2018). Electric field analyses
on monolayer semiconductors:
the example of InSe. Phys. Chem. Chem. Phys..

[50] Topsakal M., Ciraci S. (2012). Effects of static charging and exfoliation of layered
crystals. Phys. Rev. B:Condens. Matter Mater.
Phys..

[51] Xu B., Íñiguez J., Bellaiche L. (2017). Designing
lead-free antiferroelectrics for energy storage. Nat. Commun..

[52] Jiang Z., Nahas Y., Prokhorenko S., Prosandeev S., Wang D., Íñiguez J., Bellaiche L. (2018). Giant electrocaloric
response in the prototypical Pb (Mg, Nb) O 3 relaxor ferroelectric
from atomistic simulations. Phys. Rev. B.

[53] Kroemer H. (2001). Nobel Lecture:
Quasielectric fields and band offsets: teaching electrons new tricks. Rev. Mod. Phys..

[54] Roberts G., Crowell C. (1973). Capacitive effects
of Au and Cu impurity levels in
Pt-N type Si Schottky barriers. Solid-State
Electron..

[55] Simmons J. G. (1967). Poole-Frenkel
effect and Schottky effect in metal-insulator-metal systems. Phys. Rev..

[56] Esteban-Puyuelo R., Sanyal B. (2021). Role of defects in
ultrafast charge recombination in
monolayer MoS 2. Phys. Rev. B.

[57] Yang J.-H., Shi L., Wang L.-W., Wei S.-H. (2016). Non-radiative
carrier recombination
enhanced by two-level process: a first-principles study. Sci. Rep..

[58] Kong L.-J., Liu G.-H., Qiang L. (2016). Electronic
and optical properties
of O-doped monolayer MoS2. Comput. Mater. Sci..

[59] Huang Y.-T., Kavanagh S. R., Scanlon D. O., Walsh A., Hoye R. L. (2021). Perovskite-inspired
materials for photovoltaics and beyond—from design to devices. Nanotechnology.

[60] Jirauschek C., Riesch M., Tzenov P. (2019). Optoelectronic
device simulations
based on macroscopic Maxwell–Bloch equations. Adv. Theory Simul..

[61] Wang H., Zhang C., Rana F. (2015). Ultrafast
dynamics of defect-assisted
electron–hole recombination in monolayer MoS2. Nano Lett..

[62] Yuan Y., Pazos S., Li J., Tian B., Alharbi O., Zhang X., Akinwande D., Lanza M. (2025). On-chip atomristors. Mater. Sci. Eng. R Rep..

[63] Bhattacharjee S., Caruso E., McEvoy N., Ó
Coileáin C., O’Neill K., Ansari L., Duesberg G. S., Nagle R., Cherkaoui K., Gity F., Hurley P. K. (2020). Insights
into Multilevel
Resistive Switching in Monolayer MoS2. ACS Appl.
Mater. Interfaces.

[64] Fu Y., Liu E., Yuan H., Tang P., Lian B., Xu G., Zeng J., Chen Z., Wang Y., Zhou W. (2017). Gated
tuned superconductivity and phonon softening in monolayer and
bilayer MoS2. npj Quantum Mater..

[65] Garcia-Goiricelaya P., Lafuente-Bartolome J., Gurtubay I. G., Eiguren A. (2019). Long-living
carriers
in a strong electron–phonon interacting two-dimensional doped
semiconductor. Commun. Phys..

[66] Liu J., Segal D. (2020). Sharp negative differential
resistance from vibrational mode softening
in molecular junctions. Nano Lett..

[67] Shirodkar S. N., Sayou Ngomsi C. A., Dev P. (2023). Small Electron Polaron in Carbon-Doped
Cubic Boron Nitride. ACS Appl. Electron. Mater..

[68] Kirk D. M., Wang L., Kuroda M. A. (2025). Electrode-Assisted
Switching in Memristors
Based on Single-Crystal Transition Metal Dichalcogenides. ACS Appl. Mater. Interfaces.

[69] Wang Y., Wu L., Wei Z., Liu Z., Cheng P., Zhang Y., Feng B., Zhang G., Ji W., Wu K. (2022). Real-space detection and manipulation of two-dimensional quantum
well states in few-layer MoS 2. Phys. Rev. B.

[70] Boschetto G., Carapezzi S., Todri-Sanial A. (2023). Non-volatile
resistive switching
mechanism in single-layer MoS 2 memristors: insights from ab initio
modelling of Au and MoS 2 interfaces. Nanoscale
Adv..

[71] Dickinson R. G., Pauling L. (1923). The crystal structure
of molybdenite. J. Am. Chem. Soc..

