# Supplementary material for: Multiscale Modeling of the RESET Sweep in a Single-Layer MoS2 Atomristor Using Density Functional Theory
Source: ACS Omega. 2026 May 28;11(22):33086–103. doi: 10.1021/acsomega.6c02926 (PMC13261595; doi:10.1021/acsomega.6c02926)
Supplement: Supplementary file 1 [file ao6c02926_si_001.pdf]

Supporting Information for Multiscale Modeling of the RESET Sweep in a Single-Layer MoS_2 Atomristor Using Density Functional Theory

Aykut Turfanda*,† and Alessio Gagliardi*,†,‡

†*Department of Electrical Engineering, TUM School of Computation, Information and Technology, Technical University of Munich, Hans-Piloty-Straße 1, 85748 Garching, Germany*

‡*Atomistic Modeling Center (AMC) and Munich Data Science Institute (MDSI), Technical University of Munich, 85748 Garching, Germany*

E-mail: aykut.turfanda@tum.de; alessio.gagliardi@tum.de

1. Details of computational methods

Input scripts for `pp.x` for visualization of wavefunction and partial charge density calculation.

Input file 1: Post-processing script for the visualization of wavefunction of the red marked DFT band in Fig. 13 at the Γ point.

```
&INPUTPP
  prefix      = '@@PREFIX@@'      ! e.g. 'mos2'
  outdir      = '@@OUTDIR@@'      ! e.g. './output'
  plot_num    = 7
  kpoint(1)   = @@KPOINT@@         ! e.g. 1
  kband(1)    = @@KBAND@@          ! e.g. 126
/
&PLOT
  iflag         = 3
  output_format = 6
  fileout       = '@@FILEOUT@@'    ! e.g. 'mos2_rho.cube'
  nx            = 64
  ny            = 64
  nz            = 12
/
```

Input file 2: Post-processing script for the visualization of partial charge density for a certain energy range.

```
&INPUTPP
  prefix       = '@@PREFIX@@'         ! e.g. 'mos2'
  outdir       = '@@OUTDIR@@'         ! e.g. 'output'
  sample_bias  = @@SAMPLE_BIAS@@d0    ! e.g. 0.0735d0 Ry = 1 eV, which
      means Fermi level to 1 eV above the Fermi level
  plot_num     = 5
/
&PLOT
  iflag         = 3
  output_format = 6
  fileout       = '@@FILEOUT@@'       ! e.g. 'partial.cube'
/
```

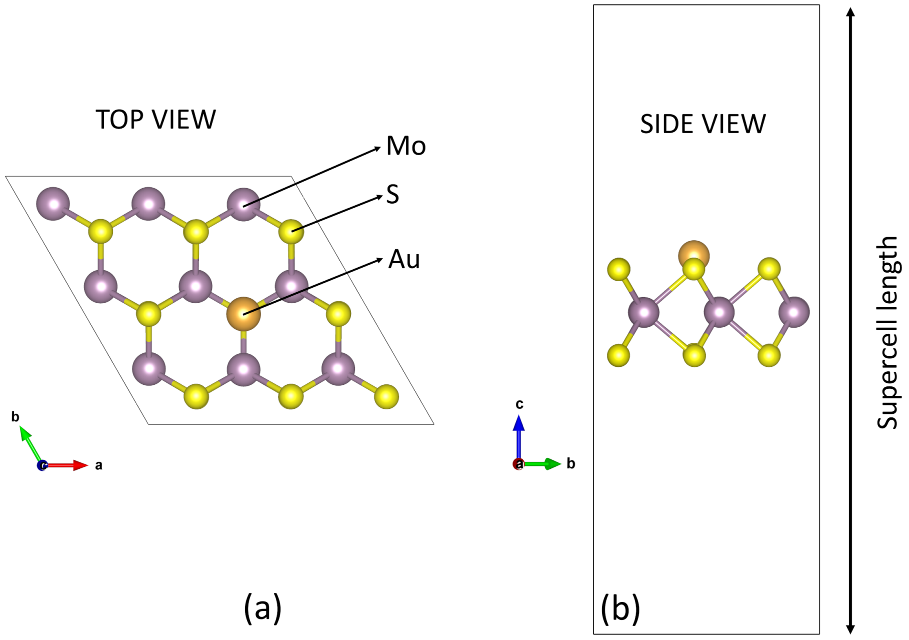

Figure S1: Atomic structure of an Au-doped (3×3) single-layer MoS_2 with its (a) top view and (b) side view, where purple balls are Mo, yellow balls are S, and the orange ball is Au.

2. Current-voltage values for the RESET sweep

If we consider the eye-reading error (when reading plots from the atomristor article) about 10% of the plot resolution, we can conclude a voltage uncertainty of plus or minus 0.05 V (tick spacing is 0.5 V) and current uncertainty with a factor of 1.26 (log-scale: $10^{0.1}$).

Table S1: Current–Voltage (I–V) data used in this study for the LRS of RESET sweep as read from the atomristor article. (The values were estimated visually from the plots. Points located midway between two minor ticks were assigned the corresponding intermediate value (for example, 1.5 between 1 and 2). If a point appeared slightly below a major tick (for example, below 2), it was approximated accordingly (for example, 1.9). All current values reported here represent approximate readings extracted from the published figures.)

Voltage (V)	Current (A)
-0.25	1.5×10^{-2}
-0.50	2.5×10^{-2}
-0.75	2.9×10^{-2}
-1.00	3.9×10^{-2}
-1.25	5.5×10^{-2}

Table S2: Current–Voltage (I–V) data used in this study for the HRS of RESET sweep as read from the atomristor article. (The values were estimated visually from the plots. Points located midway between two minor ticks were assigned the corresponding intermediate value (for example, 1.5 between 1 and 2). If a point appeared slightly below a major tick (for example, below 2), it was approximated accordingly (for example, 1.9). All current values reported here represent approximate readings extracted from the published figures.)

Voltage (V)	Current (A)
-0.25	4.0×10^{-8}
-0.50	1.5×10^{-7}
-0.75	2.0×10^{-7}
-1.00	4.0×10^{-7}
-1.25	9.0×10^{-7}

3. Additional electronic structure calculations

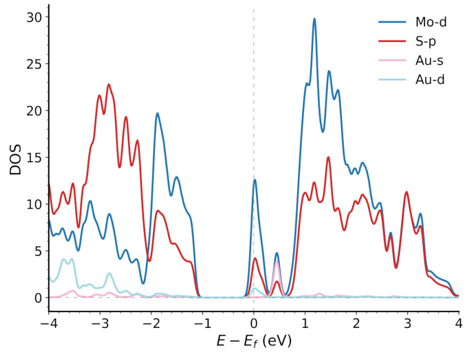

Figure S2: Projected density of states (PDOS) [eV/states/cell] for Mo's d-orbital, Mo-d, S's p-orbital, S-p, Au's s-orbital, Au-s, and Au's d-orbital, Au-d, versus E-E_f relation for Au-doped (3×3) single-layer MoS_2 at -0.02 au, where - represents the direction opposite of + in the z-axis. The electric field amplitudes are given in atomic units (au). The Fermi level is denoted by E_f.

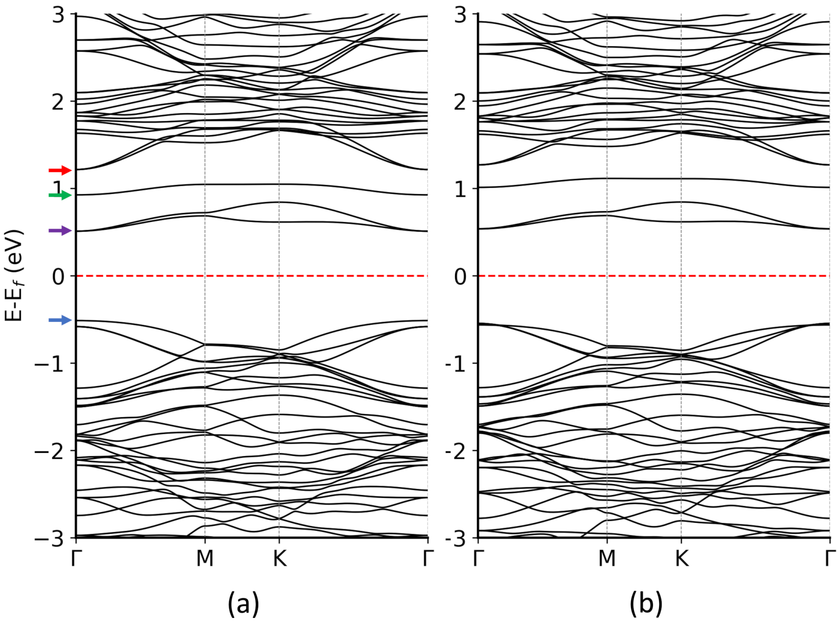

Figure S3: Electronic band structure (with numerical atomic orbital basis set) of positively-charged Au-doped (3×3) single-layer MoS_2 at electric field amplitude and direction of -1 V/Å for (a) without optimization and (b) with optimization, where - represents the direction opposite of + in the z-axis. The Fermi level is denoted by E_f. The band structure is plotted along the high-symmetry path Γ-M-K-Γ. Red, green, purple, and blue arrows represent the DFT bands with 1.21 eV, 0.92 eV, 0.50 eV, -0.51 eV energies at Γ point for panel (a) and 1.26 eV, 1.01 eV, 0.53 eV, -0.54 eV energies at Γ point for panel (b). Energies read from plots upto three significant digit.

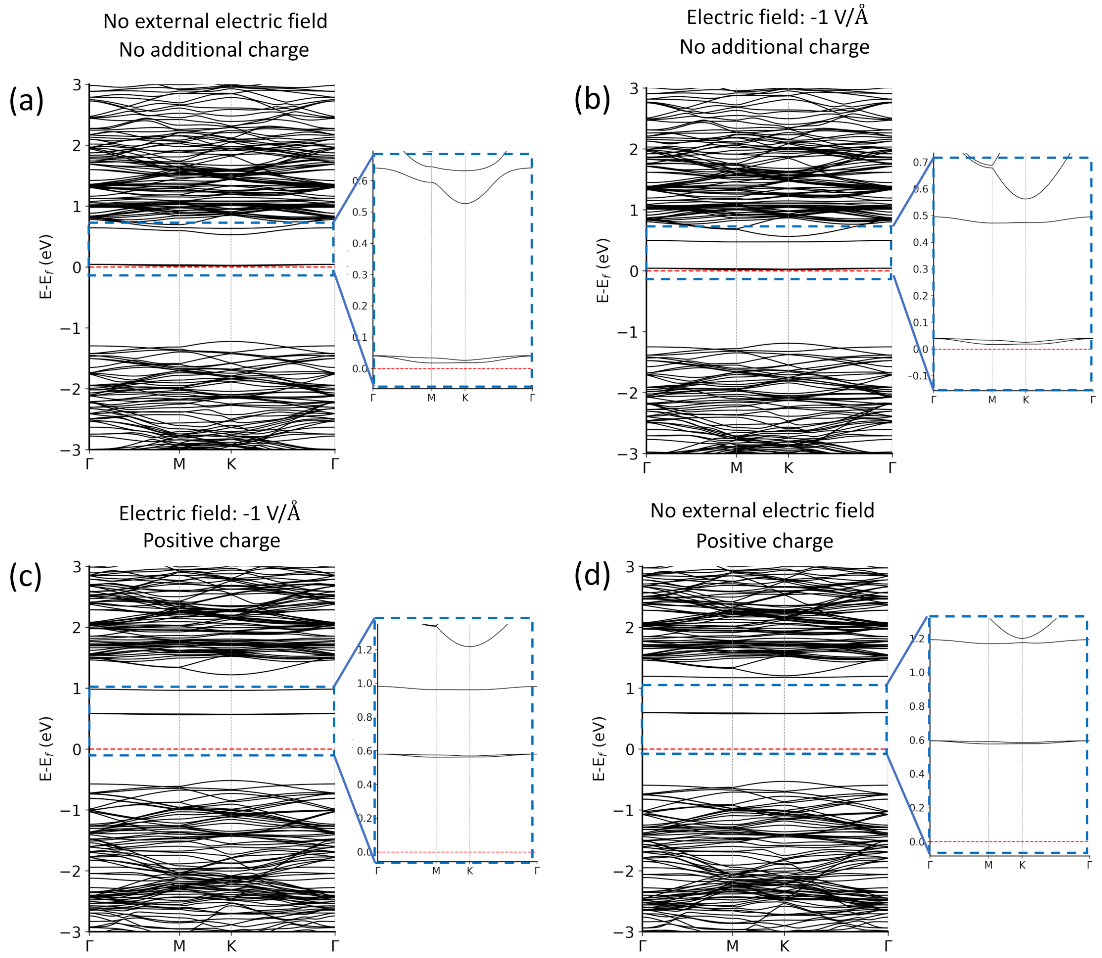

Figure S4: Electronic band structure (with numerical atomic orbital basis set) of Au-doped (5 × 5) single-layer MoS_2 (a) without external electric field and without additional charge case, (b) with external electric field (-1 V/Å) and without additional charge case, (c) with external electric field (-1 V/Å) and with additional charge case, and (d) without external electric field and with additional charge case, with slab dipole correction. Insets show the enlarged views. Negative sign, -, represents the direction opposite of + in the z-axis. The Fermi level is denoted by E_f. The band structure is plotted along the high-symmetry path Γ-M-K-Γ.

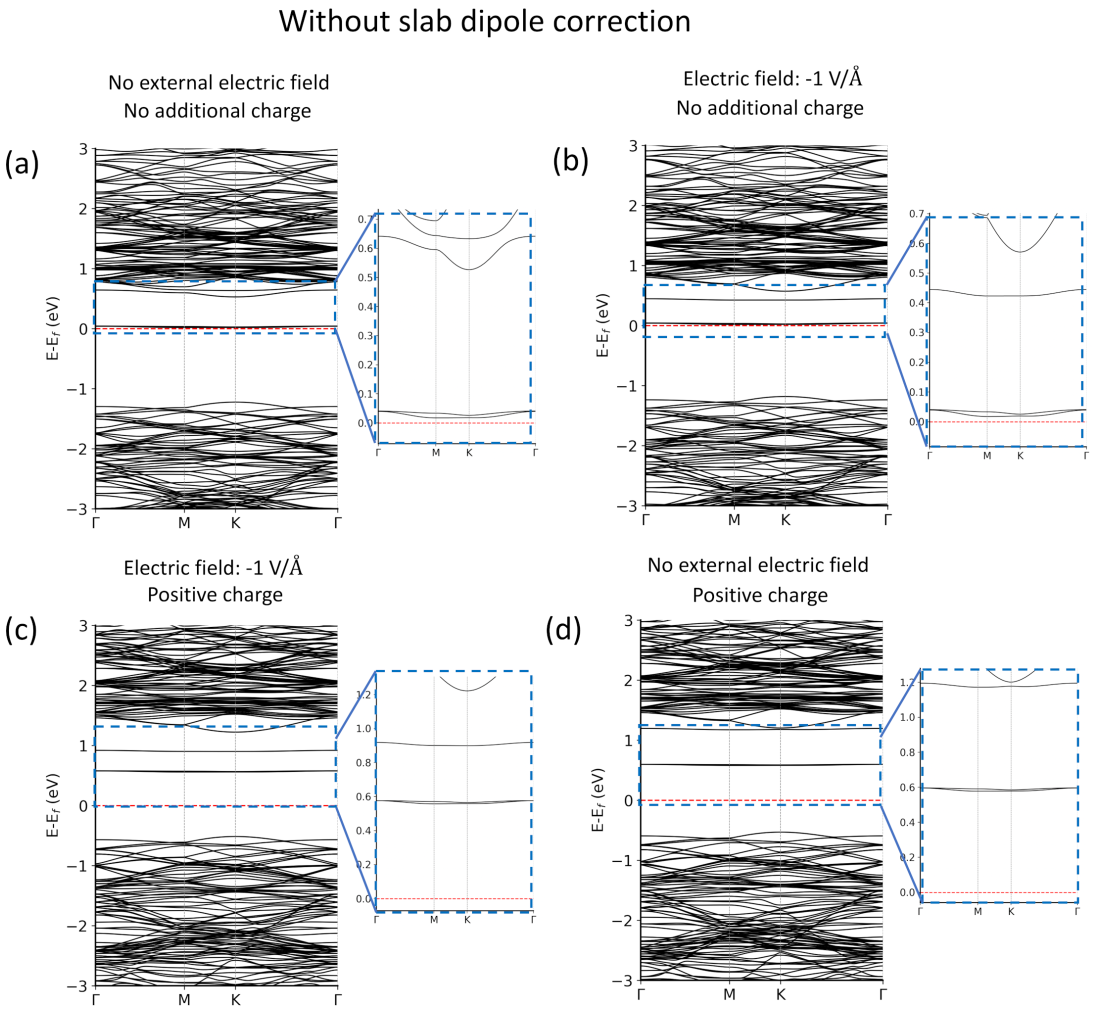

Figure S5: Electronic band structure (with numerical atomic orbital basis set) of Au-doped (5 × 5) single-layer MoS_2 (a) without external electric field and without additional charge case, (b) with external electric field (-1 V/Å) and without additional charge case, (c) with external electric field (-1 V/Å) and with additional charge case, and (d) without external electric field and with additional charge case, without slab dipole correction. Insets show the enlarged views. Negative sign, -, represents the direction opposite of + in the z-axis. The Fermi level is denoted by E_f. The band structure is plotted along the high-symmetry path Γ-M-K-Γ.

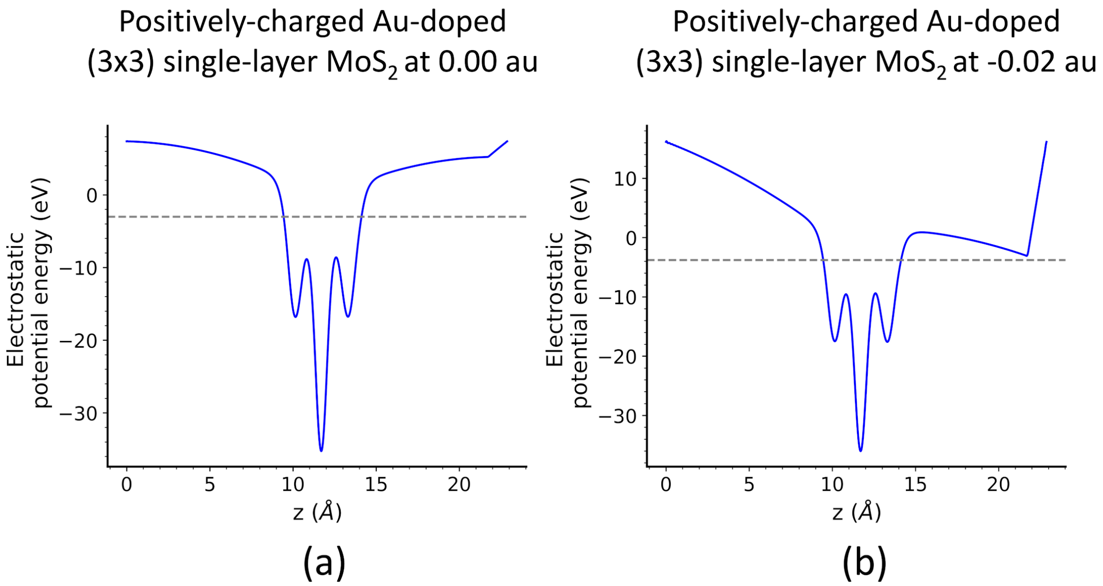

Figure S6: Electrostatic potential energy profile of (a) positively-charged Au-doped (3×3) single-layer MoS_2 at 0.00 au and (b) positively-charged Au-doped (3×3) single-layer MoS_2 at -0.02 au. Dashed gray horizontal line indicates the Fermi level. Negative sign, -, represents the direction opposite of $+$ in the z-axis.

4. Bulk Schottky barrier model

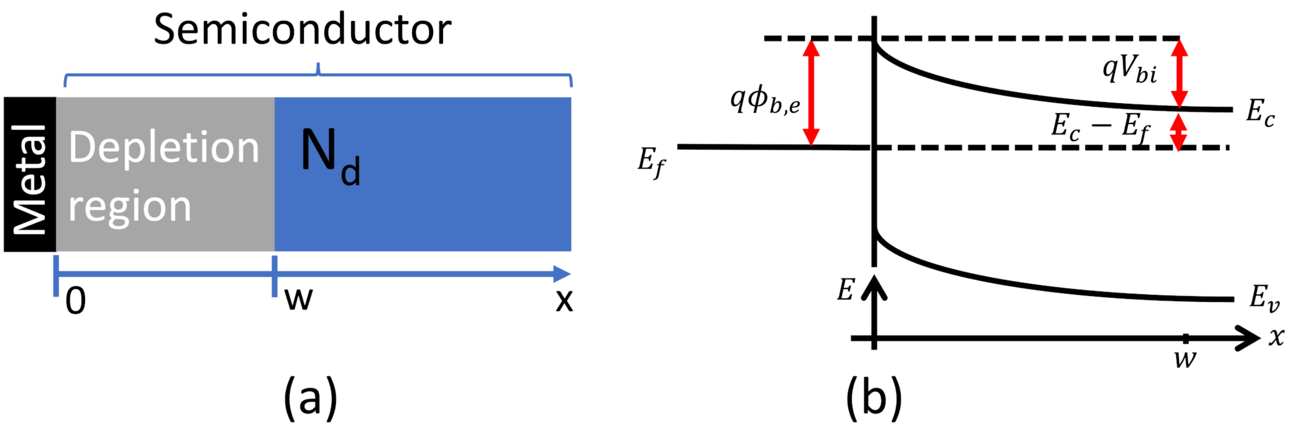

Figure S7: (a) A cartoon model of a Schottky junction in one dimension, x, with its depletion width, w, (not to scale) and (b) typical energy band diagram of a Schottky barrier model at thermal equilibrium for a metal and an n-type depletion layer semiconductor.

We consider a bulk Schottky barrier model (assuming a well-defined depletion layer) composed of Au electrode and single-layer MoS_2 as depicted in Fig. S7, where we write the Poisson equation as, $d^2\phi/dx^2 = -(qN_d)/\epsilon$, where q is the elementary electric charge, N_d is the concentration of donors, and ϵ is the permittivity and it equals to $\epsilon_{2-D,\perp} \cdot \epsilon_0$. We solve this equation by considering the following boundary conditions: (i) $\phi(x = 0) = 0$, (ii) ϕ(x = w) = V_{bi} - V, and (iii) $d\phi/dx = 0$ at x=w, where w is the depletion layer width in the semiconductor, V_{bi} is the built-in potential, and V is the applied voltage bias. The resulting $\phi(x)$ is given in Eq. S1,

$$\phi(x) = -\frac{qN_d}{2\epsilon}(x - w)^2 + V_{bi} - V, \tag{S1}$$

where w is equal to, $(2\epsilon \cdot (V_{bi} - V)/(qN_d))^{1/2}$, and V_{bi} is equal to, $\phi^*_{b,e} - V_T \cdot ln(N_c^{2-D}/N_d)$, where $\phi^*_{b,e}$ is the corrected Schottky barrier height for electrons, V_T is equal to kT/q, k is the Boltzmann constant, T is the temperature, and N_c^{2-D} is the two-dimensional effective density of states in the conduction band.[1] We consider the literature values of the above quantities as $\epsilon_{2-D,\perp} = 6.09$ and $\phi^*_{b,e} = 0.76$ eV, and we took N_c^{2-D} as equal to 9.94 $\times$ 10^{12} cm^{-2}, and m_e^* is read from the SET article of the atomristor as 0.46 m_o, where m_o is the bare electron mass.[2]

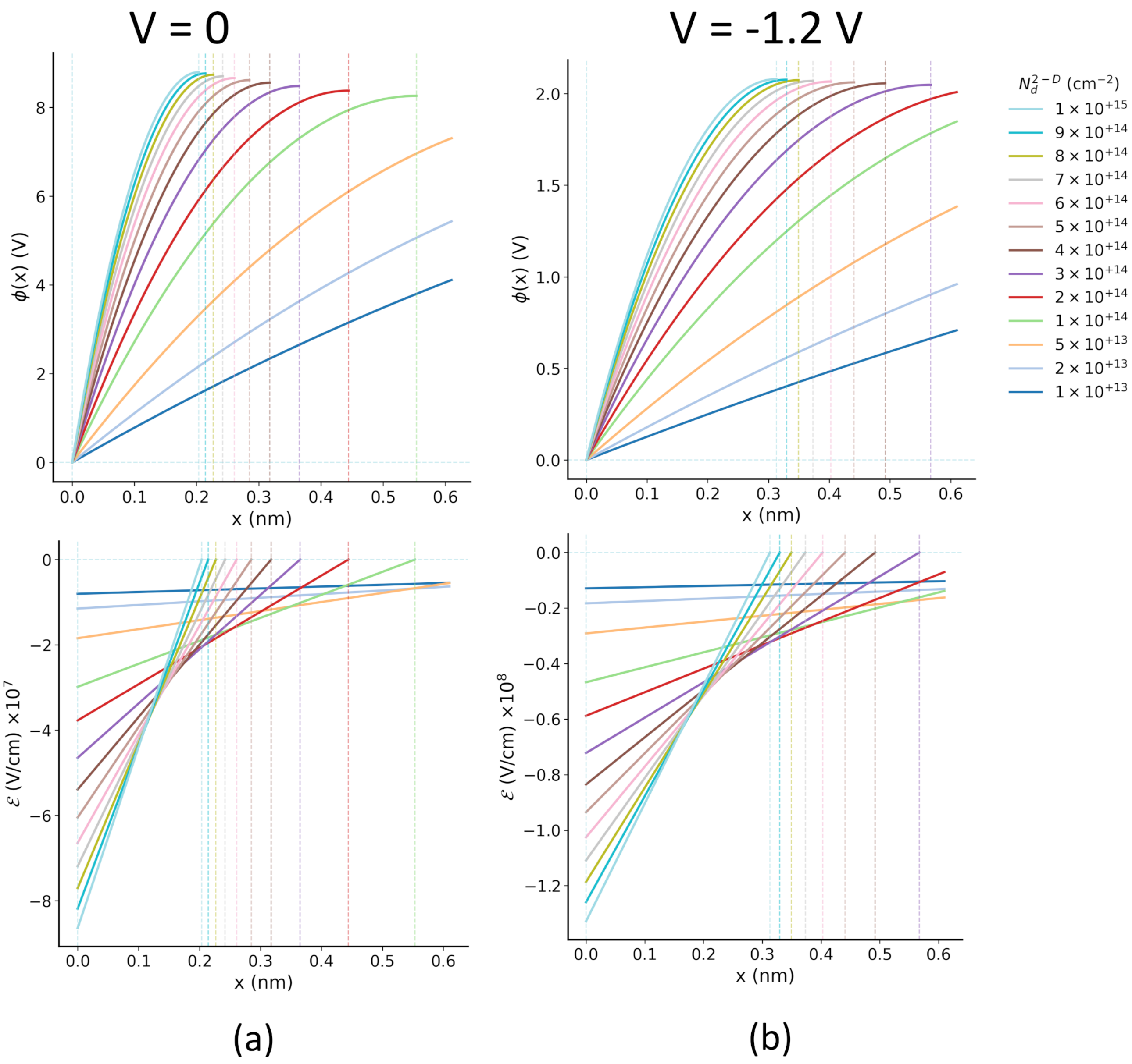

Figure S8: Electrostatic potential profile, $\phi(x)$, and electric field profile, $\mathcal{E}$, with respect to x for different dopant densities, N_d^{2-D}, for a metal and an n-type semiconductor junction at (a) V = 0 and (b) V = -1.2 V.

The solution of Eq. S1 by considering the above-mentioned parameters for a Au electrode and single-layer MoS_2 junction yields the electrostatic potential profile and electric field profile. These are depicted in Fig. S8(a) for various N_d^{2-D}s (which may also correspond to different cycles of the device operation) considering $T = 300$ K, V = 0, and N_d is equal to N_d^{2-D}/t_{MoS_2}, where t_{MoS_2} is the single-layer MoS_2 thickness. Similarly, electrostatic potential

profile and electric field profile for V = -1.2 V is given in Fig. S8(b) for various N_d^{2-D}s.

We represent the electrostatic potential profile up to w in the x-axis in Fig. S8 for each N_d^{2-D} with the grid spacing of 0.002 nm. We compute the depletion width and mark them in Fig. S8 for each N_d^{2-D} with dashed vertical lines. We observe a negative correlation between N_d^{2-D} and w as shown in Fig. S8. Moreover, the peak value of the electrostatic potential also decreases when we decrease N_d^{2-D}. These variations indicate band bending and change in charge distribution with doping in two-dimensional semiconductors. We calculate $\mathcal{E}(x)$ by differentiating -$\phi(x)$ with respect to x and we found, $\mathcal{E}(x) = (q \cdot N_d/\epsilon)(x - w)$. Then, we calculate the electric field at its maximum value; namely at x equals to zero, using $\mathcal{E}_{max} = -(q \cdot N_d/\epsilon)(w)$.

5. Poole-Frenkel barrier lowering

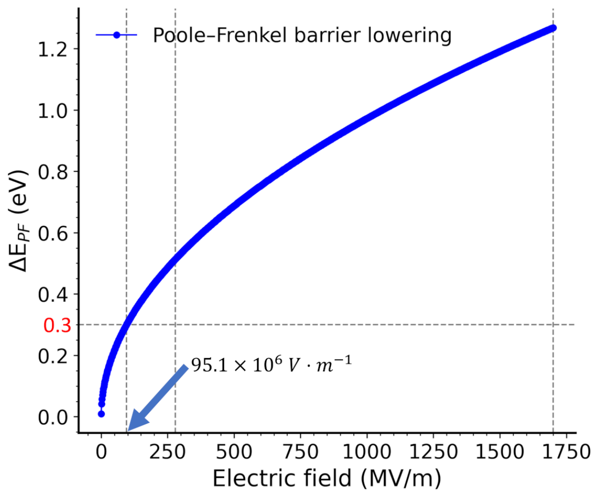

Figure S9: Poole-Frenkel barrier lowering, ΔE_{PF}, versus electric field relation.

The energy difference between the conduction band minimum and the red marked DFT band of the positively-charged Au-doped (3×3) single-layer MoS_2 at -0.02 au is equal to 0.3 eV. This is a barrier necessary need to be surpassed by field-induced Poole-Frenkel emission. Therefore, electrons at the trap states can be emitted to conduction band under electric field. We calculate the field-induced barrier lowering, ΔE_{PF}, with respect to the electric field as used for bulk materials in Fig. S9 using Eq. S2,

$$\Delta E_{PF} = \sqrt{\frac{q^3 \cdot \mathcal{E}}{\pi \epsilon_o \cdot \epsilon_{2-D,\perp}}}, \tag{S2}$$

where $\mathcal{E}$ is the electric field.[3] The electric field value, when ΔE_{PF} is equal to 0.3 eV, is found as 95.1×10^6 V·m^{-1}, which is lower than $\mathcal{E}_{max}$. Therefore, the traps can be emptied.

6. Maxwell-Bloch equations

We mark the green energy level in Fig. 13(c) as the a and the red energy level in Fig. 13(c) as the b and we assumed a two-level approximation for these two energy levels in the semi-conductor. We define the time evolution of Bloch vector components as s_1, s_2, and s_3 as given below with von Neumann equation for a two-level system under a time-dependent perturbation using the Bloch vector,

$$\frac{ds_1}{dt} = -\omega_{ba} \cdot s_2 - \gamma_b \cdot s_1, \tag{S3}$$

$$\frac{ds_2}{dt} = \omega_{ba} \cdot s_1 - 2\Omega \cos(\omega_{ba} t) \cdot s_3 - \gamma_b \cdot s_2, \tag{S4}$$

$$\frac{ds_3}{dt} = 2\Omega \cos(\omega_{ba} t) \cdot s_2, \tag{S5}$$

where t is time, w_{ba} is the system's eigenfrequency, Ω is the Rabi frequency (real part), γ_b is the phase relaxation rate and it is equal to inverse of the phase relaxation time.[4] The term s_3 is related to the population difference between level a and level b.

To visualize the time evolution of the Bloch vector components with respect to time. We picked the initial condition as $s_1 = s_2 = 0$ and $s_3 = 1$, which means that all the population is in the higher energy state. Band gap is taken as the energy difference between two energy levels as 0.51 eV and we consider a field given by $E = E_o \cos(\omega_o t)$ and it is in resonance with the two-level system. Therefore, $\hbar w_o = 0.51$ eV. We also assumed that $\gamma_a = 0$. We write the field strength, E_o, in terms of Rabi frequency as $\Omega_R = (d \cdot E_o)/ \hbar$, where d is the dipole matrix element.[4] Then, we simulate the system for $\Omega = 0.03\ \omega_o$ and $\Omega = 0.1\ \omega_o$ with time interval of $T = 8\pi/\Omega$, where Ω represent the strength of the field.

We simulate the system both at high field and at low field and we simulate it for $\gamma_b = 1/\tau$, where $\tau = 2 \times 10^{-12}$ s, and for $\gamma_b = 0$, where the carrier lifetime of single-layer MoS_2 can be read from the literature as it is varying from 1 ps to >100 ps.[5] We see Rabi oscillations in Fig. S10 and the transitions between the state a and state b is shown as a blue dashed line, s_3. These oscillations are oscillates between -1 and 1, which are representing the state

a in its fully occupied case and the state b in its fully occupied case. We saw that the population difference oscillates in Fig. S10. In case of γ_b not equal to 0, we saw that s_3's amplitude decreases over time. Similarly, in case γ_b is equal to 0, we saw that s_3's amplitude is constant over time. At high field strength, Ω, we have more oscillations in a given time interval compared to low field strength.

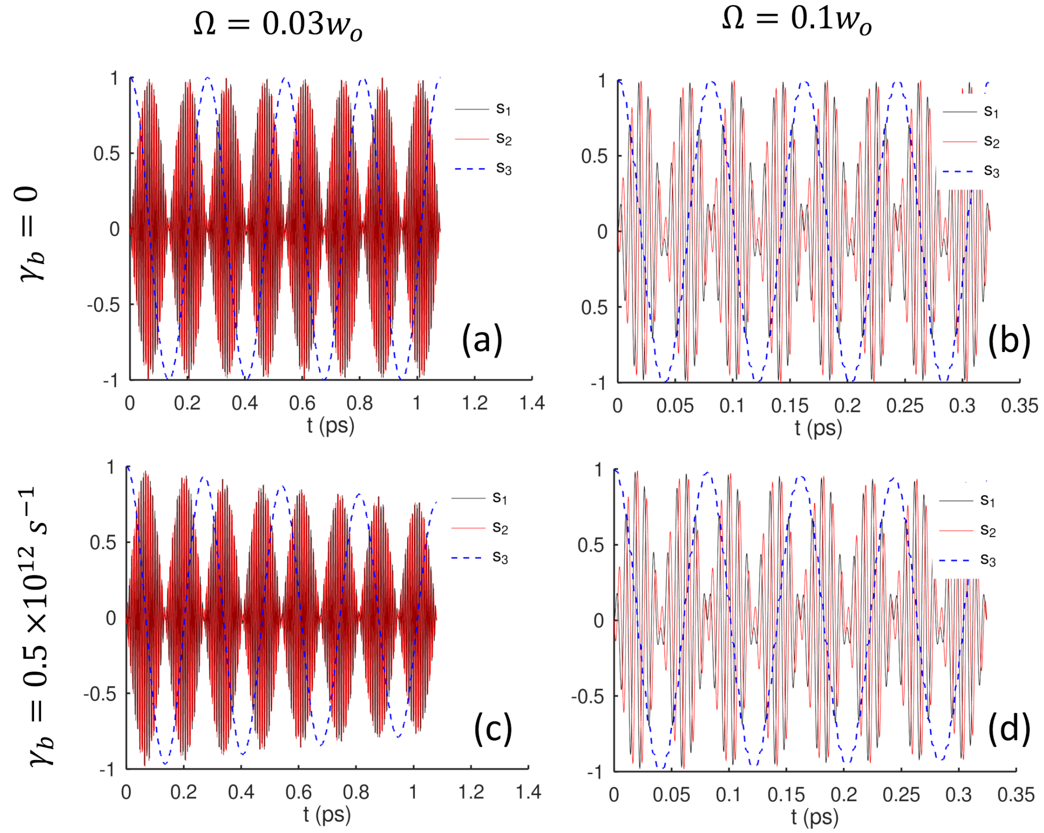

Figure S10: Variation of s_1, s_2, and s_3 with respect to time for (a) $\gamma_b = 0$ and $\Omega = 0.03\ \omega_o$, (b) $\gamma_b = 0$ and $\Omega = 0.1\ \omega_o$, (c) $\gamma_b = 0.5 \times 10^{12}$ s^{-1} and $\Omega = 0.03\ \omega_o$, and (d) $\gamma_b = 0.5 \times 10^{12}$ s^{-1} and $\Omega = 0.1\ \omega_o$, where γ_b is the phase relaxation rate and Ω is the Rabi frequency.

References

(1) *Physics of Semiconductor Devices*; John Wiley & Sons, Ltd, 2006; pp 134–196.

(2) Turfanda, A.; Gagliardi, A. Single-Layer MoS2-Based Atomristor's Resistive Switching Model for SET Sweep with Density Functional Theory Simulations. *ACS Applied Electronic Materials* **2025**,

(3) Simmons, J. G. Poole-Frenkel effect and Schottky effect in metal-insulator-metal systems. *Physical Review* **1967**, *155*, 657.

(4) Jirauschek, C.; Riesch, M.; Tzenov, P. Optoelectronic device simulations based on macroscopic Maxwell–Bloch equations. *Advanced Theory and Simulations* **2019**, *2*, 1900018.

(5) Wang, H.; Zhang, C.; Rana, F. Ultrafast dynamics of defect-assisted electron–hole recombination in monolayer MoS2. *Nano letters* **2015**, *15*, 339–345.
